# Core–shell NaBH_4_@Ni Nanoarchitectures: A Platform for Tunable Hydrogen Storage

**DOI:** 10.1002/cssc.202200664

**Published:** 2022-07-13

**Authors:** Muhammad Saad Salman, Yuwei Yang, Muhammad Zubair, Nicholas M. Bedford, Kondo‐Francois Aguey‐Zinsou

**Affiliations:** ^1^ MERLin School of Chemical Engineering The University of New South Wales Sydney NSW 2052 Australia; ^2^ School of Chemical Engineering The University of New South Wales Sydney NSW 2052 Australia; ^3^ MERLin School of Chemistry The University of Sydney Sydney NSW 2006 Australia

**Keywords:** borohydrides, core–shell, hydrides, hydrogen storage, nanostructures

## Abstract

The core–shell approach has surfaced as an attractive strategy to make complex hydrides reversible for hydrogen storage; however, no synthetic method exists for taking advantage of this approach. Here, a detailed investigation was undertaken to effectively design freestanding core–shell NaBH_4_@Ni nanoarchitectures and correlate their hydrogen properties with structure and chemical composition. It was shown that the Ni shell growth on the surface of NaBH_4_ particles could be kinetically and thermodynamically controlled. The latter led to varied hydrogen properties. Near‐edge X‐ray absorption fine structure analysis confirmed that control over the Ni^0^/Ni_*x*_B_*y*_ concentrations upon Ni^II^ reduction led to a destabilized hydride system. Hydrogen release from the sphere, cube, and bar‐like core–shell nanoarchitectures occurred at around 50, 90, and 95 °C, respectively, compared to the bulk (>500 °C). This core–shell approach, when extended to other hydrides, could open new avenues to decipher structure–property correlation in hydrogen storage/generation.

## Graphical abstract

**The core–shell opportunity**: Freestanding core–shell NaBH_4_@Ni nanoarchitectures are designed by optimizing a simple wet‐chemistry approach. Improved hydrogen properties can be achieved by adjusting Ni deposition on the surface of NaBH_4_ particles and the resulting Ni^0^/Ni_*x*_B_*y*_ concentration.

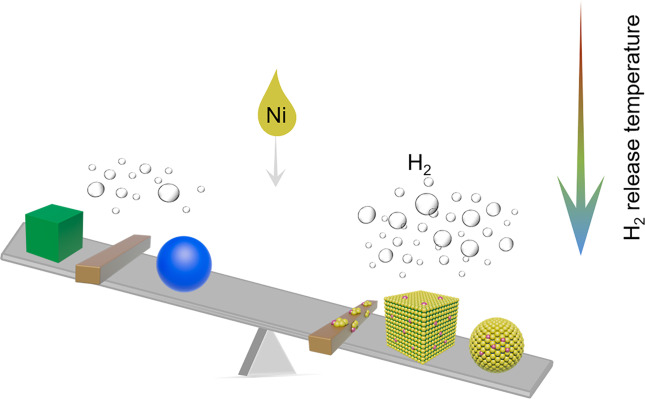

## Introduction

Hydrogen is a potential clean energy vector, often touted as the fuel of the future. Hydrogen can be made from renewables and remains an important component of many industrial processes.^[1]^ However, the storage of hydrogen in a safe and compact form with high energy density is difficult. Owing to the possible high energy density, materials‐based solid‐state hydrogen storage has remained at the center of many research efforts.^[2]^ Among potential hydrogen storage materials, borohydrides are ideal for hydrogen storage due to their high gravimetric densities.^[3]^ Particularly, sodium borohydride (NaBH_4_) is a preferred choice for solid‐state hydrogen storage due to its high hydrogen content of around 10.6 mass % H_2_, stability in the air, ease in handling, and hydrogen release in a single step upon heating.[^3a^, ^3c^] However, in its pristine state, NaBH_4_ requires a very high temperature (≈500 °C) for hydrogen release due to its high enthalpy of decomposition, and the reversible uptake of hydrogen under moderate conditions of temperature and pressure is difficult mainly because of the loss of Na and B.^[4]^

To date, several strategies have been proposed to destabilize NaBH_4_ and enable H_2_ release at lower temperatures and hydrogen reversibility. These include (i) partial substitution of Na by additives,^[5]^ (ii) formation of reactive hydrides,^[6]^ and (iii) nanoconfinement in porous scaffolds.^[7]^ All of these strategies weaken the B−H bonds in NaBH_4_ and improve H_2_ properties. Among them, nanoconfinement of NaBH_4_ is attractive because it has the potential to lead to improvements in the H_2_ kinetics and thermodynamics, while keeping the dehydrogenation products in close vicinity and “active” state for rehydrogenation. Indeed, nanoconfinement of LiBH_4_ or NaBH_4_ into nanoporous carbons,[^7a^, ^8^] hollow CuS,^[9]^ SiO_2_,[^8^, ^10^] and/or graphene^[11]^ scaffolds have been reported to lead to lower H_2_ release temperatures with significant improvements in hydrogen reversibility and faster (de)hydrogenation kinetics. However, these nanoconfinement approaches suffer from a range of drawbacks such as the dead mass of the scaffolds, leading to low H_2_ volumetric and gravimetric capacity for the confined hydride. The confined hydride has also been shown to leach out from the pores of the scaffolds upon H_2_ cycling.

An alternative is to confine freestanding hydride nanostructures, for example, through a core–shell approach whereby the hydride core is confined within a shell acting as a gateway for facilitating H_2_ uptake/release while keeping the dehydrogenation products in close vicinity and avoiding elemental loss.[^3b^, ^12^] A significant advantage of this approach is that it allows for tuning the H_2_ properties of hydrides through shape/morphology control. Indeed, shape‐ or structure‐dependent H_2_ release/uptake correlations have been observed in nanostructures of Pd, where a high number of vertices and (100) facets were found to accelerate H_2_ release/uptake compared to (111) facets.^[13]^ In another work, SiO_2_ stabilized rhombic dodecahedral Pd nanoparticles enclosed by (110) facets showed higher H_2_ release per unit area compared to the regular Pd cubes, cubes with protruded edges, and branched nanoparticles, due to the difference in their exposed surface planes.^[14]^ Similarly, Kitagawa and co‐workers reported that Pd cubes covered with MOF [copper(II) 1,3,5‐benzenetricaboxylate] showed up to around 75 % increase in H_2_ uptake capacity compared to the uncovered Pd cubes.^[15]^

Freestanding borohydride nanoparticles can be synthesized by using stabilizers such as polymers and surfactants. For example, LiBH_4_ spherical nanoparticles of size around 10 nm could be formed with poly(methyl methacrylate) (PMMA) as a capping agent, and this led to a significant H_2_ release at around 70 °C compared to the bulk, which releases H_2_>450 °C.^[16]^ The same approach was extended to PMMA‐capped NaBH_4_ and Ca(BH_4_)_2_ nanostructures. The formation of LiBH_4_ nanobelts with a width of around 10–40 nm upon the coordination of diethyl ether was also reported to occur during ball milling.^[17]^ The possibility of controlling the size and nanoarchitecture of LiBH_4_ and/or NaBH_4_ with surfactants^[18]^ or counter ions^[19]^ has also been reported. However, methods for effectively growing a metallic shell on the surface of freestanding borohydride nanoarchitectures have received less attention because of the difficulty in controlling the nucleation and growth parameters. For example, Christian and Aguey‐Zinsou reported the synthesis of the core–shell NaBH_4_@M (M=Co, Cu, Ni, Sn) structures by reducing metal precursors directly on the surface of the surfactant stabilized NaBH_4_ nanoparticles in solution.^[20]^ In the case of NaBH_4_@Ni, owing to a higher H_2_ permeability of Ni compared to other metals, a reversible H_2_ capacity of around 5 mass % was achieved at 350 °C, 4 MPa H_2_ for 5 cycles.[^20^, ^21^] These preliminary findings suggest that the structure–hydrogen property correlations from NaBH_4_@Ni nanoarchitectures would be possible once the nucleation and growth parameters are well controlled and understood. As a result, the growth mechanisms that favor nucleation and growth of the shell on the surface of the borohydride cores are influenced by a variety of factors such as non‐coordinating solvent, coordinating ligands, seed/shell precursor ratios, type of the shell precursor and its reduction kinetics, reaction time, and/or temperature.[^20^, ^21^, ^22^]

Herein, using NaBH_4_ as a model hydride, we report on a simple wet‐chemistry based approach to design freestanding sphere, cube, and bar‐like core–shell NaBH_4_@Ni nanoarchitectures. The surfactant‐directed NaBH_4_ nanoarchitectures were obtained by using tetrabutylammonium bromide (TBAB), octadecylamine (ODA), and tridecanoic acid (TDA),[^18a^, ^23^] and coated with Ni by using the Ni‐oleylamine (Ni‐OAm) complex. Tuning the parameters for homogeneous and heterogeneous nucleation allowed us to control the amount of Ni‐OAm needed for the growth of the Ni shell over different NaBH_4_ shapes. Structural investigations revealed that the nucleation and growth of the Ni shell are sensitive to the temperature, deposition time, and atomic diffusion of Ni^II^ ions on the surface of NaBH_4_. Despite the high reactivity of NaBH_4_, kinetic and/or thermodynamic control over NaBH_4_@Ni growth was possible in the presence of ligands. Further investigations using near‐edge X‐ray absorption fine structure (NEXAFS) revealed that proper tuning of Ni^0^ and Ni_*x*_B_*y*_ species in NaBH_4_@Ni may lead to improved hydrogen release properties compared to bulk NaBH_4_.

## Results and Discussion

### Synthesis of core–shell NaBH_4_@Ni nanoarchitectures

**Figure 1:**
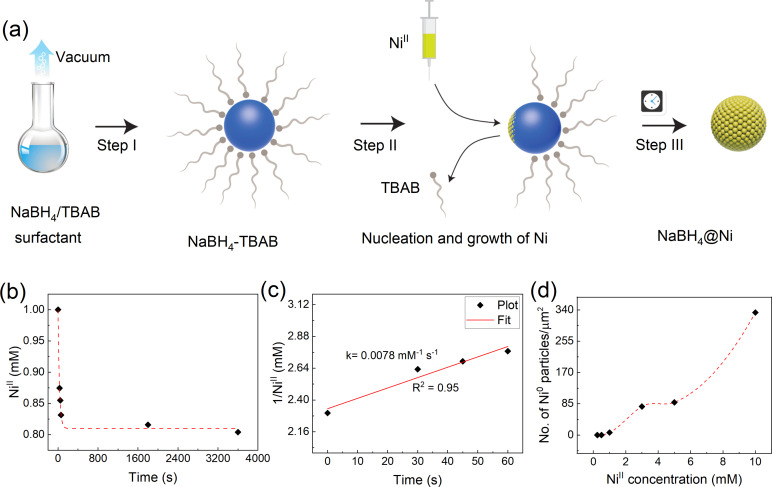
(a) Schematic illustration of the synthesis of NaBH_4_‐TBAB nanoparticles via solvent evaporation and their core–shell NaBH_4_‐TBAB@Ni structure using Ni‐OAm complex. (b) Reduction kinetics of Ni‐OAm (normalized concentration) with NaBH_4_‐TBAB particles suspended in toluene at RT. The concentration of Ni^II^ remaining in the solution was measured by ICP‐MS at different intervals. (c) Experimental data fit integrated to the second‐order rate law (1/[Ni^II^]=*kt*+1/[Ni^II^]_0_). (d) Number of Ni^0^ particles obtained at different concentrations of Ni‐OAm with 50 mg mL^−1^ of NaBH_4_‐TBAB particles in toluene at RT. The number of Ni^0^ per unit μm^2^ was estimated from different transmission electron microscopy (TEM) images (not shown).

The approach advanced here involves a simple two‐step synthetic process where different (sphere, cube, and/or bar‐like) NaBH_4_ nanoarchitectures suspended in solution are coated with Ni. Briefly, the NaBH_4_ nanoarchitectures of controlled morphologies were first synthesized by evaporating a solution containing dissolved NaBH_4_ and the respective surfactant (e.g., TBAB, Figure 1a, step I).^[23]^ Second, the TBAB‐stabilized NaBH_4_ particles were coated with Ni by injecting the Ni‐OAm complex at a given rate to control the deposition and reduction of Ni^II^ on the surface of the NaBH_4_ particles suspended in toluene (Figure 1a, step II). The solution was then aged before separation and dried to get the sphere‐like core–shell NaBH_4_‐TBAB@Ni (Figure 1a, step III). A similar procedure was used for NaBH_4_‐ODA and NaBH_4_‐TDA to synthesize the core–shell NaBH_4_‐ODA@Ni (cube‐like) and NaBH_4_‐TDA@Ni (bar‐like) nanoarchitectures. Herein, the Ni‐OAm complex was used instead of commercially available anhydrous Ni salts (e.g., NiCl_2_) because the latter has poor solubility in organic media (e.g., toluene, cyclohexane, and/or THF; Figure S1).

During the coating process, NaBH_4_ also acts as the reducing agent for Ni^II^ due to a large difference in the standard reduction potential of NaBH_4_ (−1.24 V) and Ni^II^/Ni^0^ (−0.23 V).^[20]^ Thus, it is expected that Ni‐OAm will be reduced to Ni^0^ in the presence of NaBH_4_‐TBAB in solution. The reduction of Ni‐OAm in solution shows that around 20 % of Ni‐OAm was consumed by NaBH_4_‐TBAB within 25 min of injection (Figure 1b). In this case, the reduction kinetics were fitted to the second‐order rate law with a rate constant (*k*) of around 0.0078 mm ^−1^ s^−1^ (Figure 1c). A second‐order law, where the rate of reduction depends only on the concentration of Ni^II^, is expected because of the excess of NaBH_4_ compared to Ni‐OAm in the solution. However, the estimated rate constant implies a slower reduction process than the instantaneous reductions commonly observed for Ni precursors.^[24]^ The slower reduction in the present work may be due to the strong interactions between NaBH_4_ and TBAB as highlighted in our previous work.^[23]^

### Growth of Ni on the surface of NaBH_4_ nanoarchitectures

In principle, there are two different pathways for the Ni atoms to nucleate in solution: homogeneous and heterogeneous nucleation. The former involves the presence of a supersaturated concentration of free Ni atoms, which coalesce and serve as nuclei for further growth. In contrast, the latter involves the deposition and reduction of Ni^II^ on the locally available NaBH_4_ surfaces owing to the lower free energy barrier for heterogeneous nucleation compared to homogeneous nucleation.^[25]^ To facilitate the nucleation and growth of Ni on the NaBH_4_ seeds, the homogeneous nucleation of Ni in solution must be avoided. The concentration of Ni‐OAm was varied to monitor the generation of isolated Ni^0^ particles in the presence of NaBH_4_‐TBAB to establish the parameters that would favor heterogeneous nucleation. Figure 1d shows the number of isolated Ni^0^ particles observed outside the NaBH_4_ seeds at different concentrations of Ni‐OAm. Isolated Ni particles were not observed between 0.25–0.5 mm of Ni‐OAm; however, at higher concentrations both homogeneous and heterogeneous nucleation are likely to occur. From these results, it can therefore be considered that 0.5 mm corresponds to the maximum concentration of Ni‐OAm that should be used to avoid the homogeneous nucleation of Ni in solution. In addition, we found that the addition of 50 μm of trioctylphosphine (TOP) with Ni‐OAm was essential to stabilize Ni on the surface of the NaBH_4_ seeds because this facilitated the attachment of Ni^II^ precursor on the surface of the NaBH_4_ particles (Figures 1d and S2).

**Figure 2:**
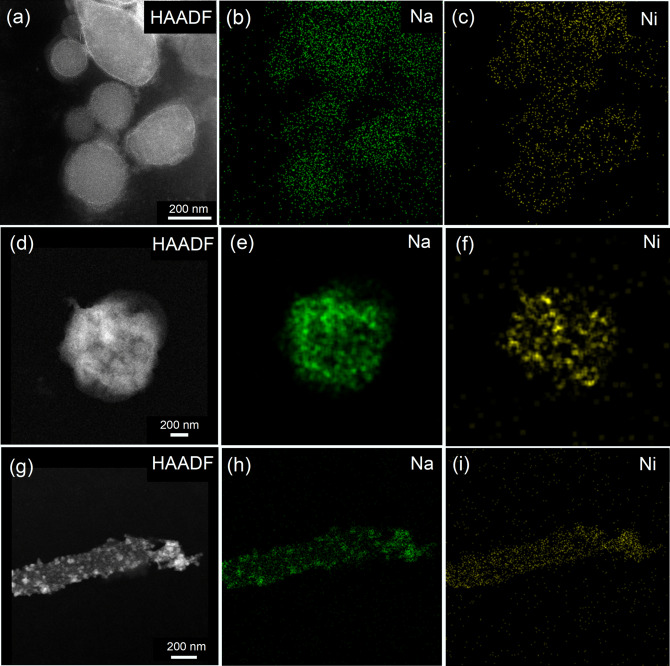
HAADF‐STEM images (scale bar: 200 nm) with Na and Ni distributions in (a–c) NaBH_4_‐TBAB@Ni, (d–f) NaBH_4_‐ODA@Ni, and (g–i) NaBH_4_‐TDA@Ni. For (g), an island growth of the Ni particles can be seen. HAADF images (a, d, g) show bright Ni particles over the lighter NaBH_4_ cores. Note that the size of the particles looks bigger and deformed in HAADF images due to the decomposition/swallowing caused by the electron beam while scanning.

The core–shell structure was further investigated by high‐angle annular dark‐field scanning transmission electron microscopy (HAADF‐STEM) analysis, which showed bright Ni particles (higher atomic number) over the lighter NaBH_4_ cores (lower atomic number, Figure 2a, d, g). This was further evidenced by elemental mapping, which displays evenly distributed Ni on the surface of the NaBH_4_ particles (Figure 2c, f, i) and the line‐scan analysis (Figure S3). Indeed, the line‐scan analysis further evidenced the formation of a Ni shell on the surface of the NBH_4_ particles (Figure S3b). A representative core–shell particle was deliberately decomposed under the electron beam to further confirm the formation of the Ni shell over NaBH_4_; as expected, a uniform Ni shell (dark) was obtained after the decomposition of the NaBH_4_ core (Figure S4). Further detailed analysis using the high‐resolution (HR)TEM and the fast‐Fourier transform (FFT) of the selected areas revealed *d*‐spacings corresponding to the Ni (111) and Ni (110) facets.^[26]^ Therefore, these results accompanied by the HAADF‐STEM and line‐scan analyses confirm the successful formation of the core–shell NaBH_4_@Ni structure.

**Figure 3:**
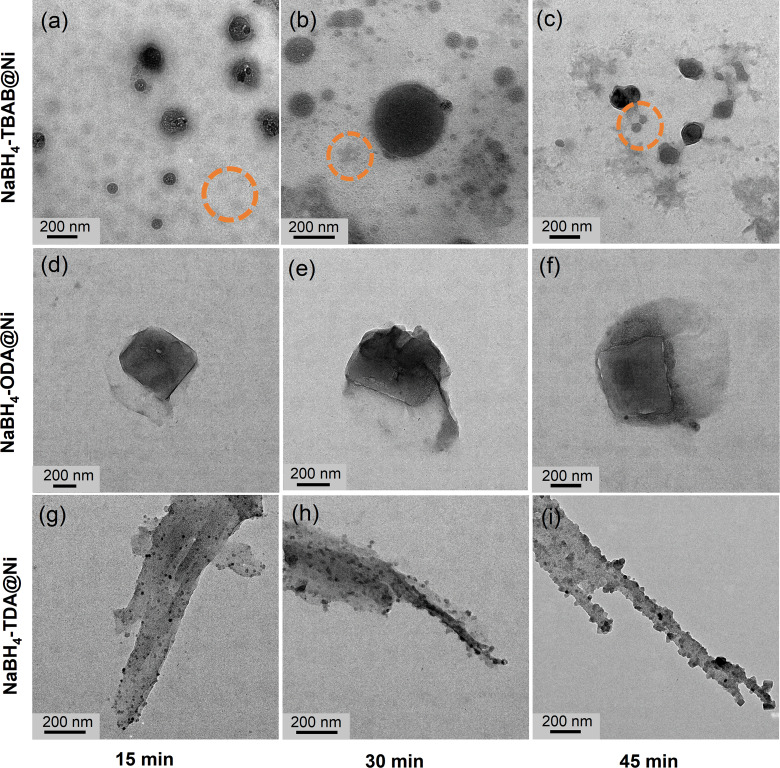
TEM images show different core–shell nanoarchitectures of (a–c) NaBH_4_‐TBAB@Ni, (d–f) NaBH_4_‐ODA@Ni, and (g–i) NaBH_4_‐TDA@Ni obtained after 15, 30, and 45 min of Ni‐OAm injection at 40 °C. Additional TEM images for NaBH_4_‐TBAB@Ni, NaBH_4_‐ODA@Ni, and NaBH_4_‐TDA@Ni are displayed in Figures S5–S7, and coated and uncoated particles are also identified for NaBH_4_‐TBAB@Ni.

To better understand the formation of the NaBH_4_@Ni nanoarchitectures, the progress of the Ni shell growth during the Ni‐OAm injections was monitored by taking a fraction of the reacting solution at different time intervals and studying the morphological evolution of the core–shell structures (Figure 3). For NaBH_4_‐TBAB@Ni, the formation of the core–shell particles was observed after 15 min of Ni‐OAm injection. Some light particles (circled in orange) of around 40–60 nm were also observed, and these could correspond to uncoated NaBH_4_ particles (herein termed as primary NaBH_4_) in solution (Figure 3a). The presence of the primary NaBH_4_ particles suggests that the deposition of Ni did not progress uniformly across all the suspended NaBH_4_‐TBAB particles, and this may be attributed to the initial low concentration of Ni‐OAm in solution. After 30 and 45 min, more core–shell particles (size ≈80–200 nm) were observed by TEM because of an increased concentration of Ni‐OAm (Figure 3b, c). During these intervals, the presence of the primary NaBH_4_ particles implies that they are the precursors of the growing core–shell particles (orange circles in Figure 3b, c). This process resembles the growth processes observed for several metals and oxide‐based core–shell nanostructures.^[27]^ A similar growth mechanism was observed for NaBH_4_‐ODA@Ni, as the light Ni shell was observed at the edges of the cube‐like particle after 15 min of Ni‐OAm injection (Figure 3d). After 30 and 45 min, with the increase in Ni‐OAm concentration, more Ni shells can be seen surrounding the cube‐like NaBH_4_‐ODA@Ni particles (Figures 3e, f and S6). A discernible layer surrounding the cube‐like NaBH_4_‐ODA@Ni particles could be due to the decomposition under the electron beam or slight consumption of the borohydride surface by amine.^[28]^

For NaBH_4_‐TDA@Ni, a few small Ni particles on the bar‐like NaBH_4_ were observed after 15 min of Ni‐OAm injection (Figure 3g), and the density of Ni^0^ particles on the bar‐like structures increased after 30–45 min (Figures 3h, i and S7). The increase in the density of discrete Ni^0^ particles on NaBH_4_‐TDA suggests the progressive deposition and reduction of Ni‐OAm on the surface of NaBH_4_ along a different growth mechanism favoring a non‐epitaxial growth of Ni^0^. Hence, it is apparent that different growth modes exist in the nucleation and growth of the Ni shells on the surface of NaBH_4_‐TBAB, NaBH_4_‐ODA, and NaBH_4_‐TDA. For all the core–shell nanoarchitectures, the morphologies of the initial NaBH_4_ nanostructures did not change significantly. This may be the result of the stabilizing surfactants guiding the growth of the Ni shell. In contrast, Ni coating on the non‐surfactant stabilized NaBH_4_ particles led to irregular structures (Figure S8).

### Nature of the shell in NaBH_4_@Ni nanoarchitectures

**Figure 4:**
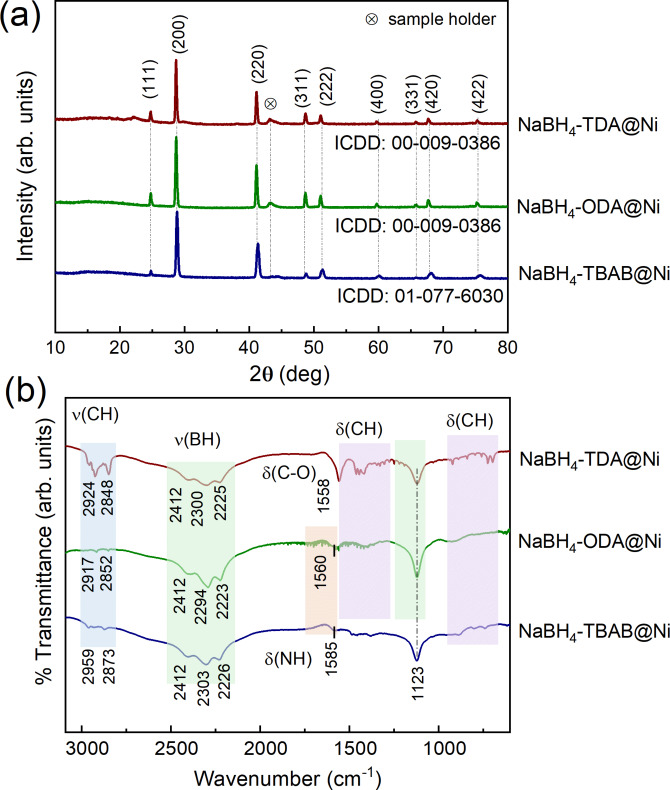
(a) XRD patterns and (b) FTIR spectra of NaBH_4_@Ni nanoarchitectures. The XRD patterns in (a) were indexed with the reference patterns retrieved from the International Centre for Diffraction Data (ICDD). The XRD pattern and FTIR spectrum of pristine NaBH_4_ are shown in Figures S9 and S10, respectively.

X‐ray diffraction (XRD) analysis of the core–shell NaBH_4_@Ni nanoarchitectures revealed that these materials remained crystalline, with the main phase corresponding to the cubic α‐phase of NaBH_4_ with no apparent traces of surfactants (Figures 4a, S9, and S11–S13). Also, no diffraction peaks corresponding to Ni^0^, its oxide and/or boride phases were observed. This could be because of their extremely small sizes of Ni particles and/or their high dispersion at the surface of the NaBH_4_ particles (rather than the formation of separate phases). It is also worth mentioning that the intensity in the XRD patterns corresponding to the NaBH_4_ phase after Ni coating was significantly reduced compared to the uncoated materials, suggesting an encapsulation of the NaBH_4_ particles by the Ni shells (Figures S11–S13).^[29]^

Fourier‐transform infrared (FTIR) analysis of the as‐synthesized core–shell nanoarchitectures showed B−H stretching and bending vibrations between 2412–2223 cm^−1^ and at 1123 cm^−1^, respectively (Figure 4b). Notably, the B−H stretching vibrations at 2390–2223 cm^−1^ of pristine NaBH_4_ were all shifted to higher wavenumbers for all the NaBH_4_@Ni nanoarchitectures indicating a strengthening of the B−H bonds after Ni coating (Figures 4b and S10).^[30]^ The additional stretching and bending vibrations between 2959–2848 cm^−1^ (C−H) and 1585–1558 cm^−1^ (−NH/−CO), respectively, indicated that traces of surfactants were left in the NaBH_4_@Ni nanoarchitectures (Figure 4b).

**Figure 5:**
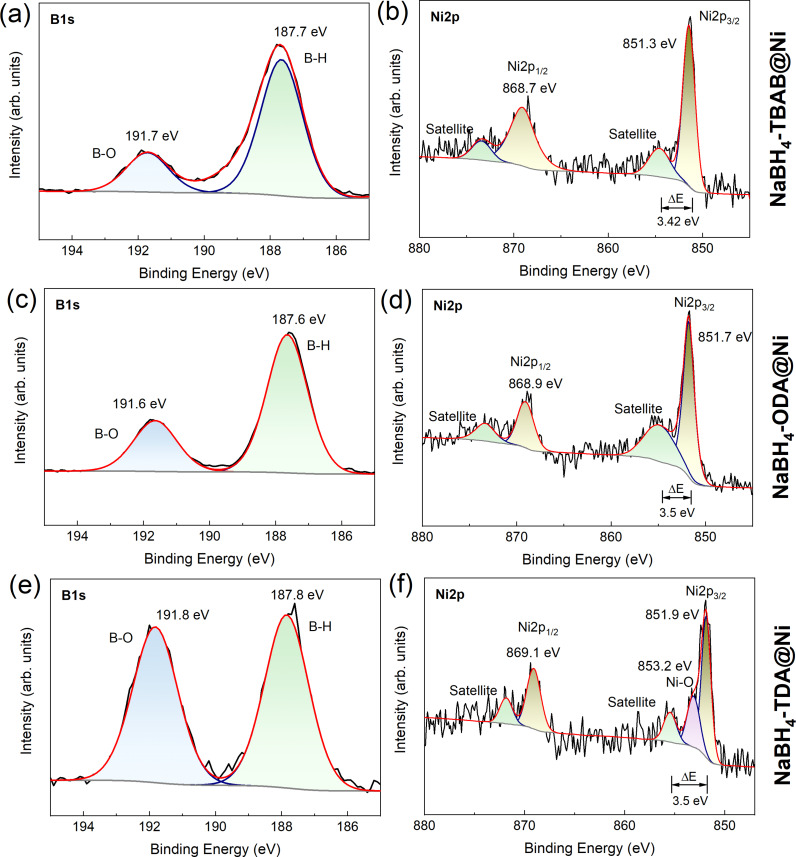
High‐resolution XPS spectra (B 1s and Ni 2p) of (a,b) NaBH_4_‐TBAB@Ni, (c, d) NaBH_4_‐ODA@Ni, and (e, f) NaBH_4_‐TDA@Ni.

The surface chemistry and electronic states of the NaBH_4_@Ni nanoarchitectures were further determined by X‐ray photoelectron spectroscopy (XPS) and compared with bulk NaBH_4_ (Figures 5 and S14–S17). For NaBH_4_‐TBAB@Ni, the B 1s peaks corresponding to B−O and B−H were observed at 191.7 and 187.7 eV, respectively (Figure 5a). For NaBH_4_‐ODA@Ni, the B−O and B−H peaks slightly shifted toward lower binding energy values at 191.6 and 187.6 eV, respectively (Figure 5c). In the case of NaBH_4_‐TDA@Ni, these peaks shifted to 191.8 and 187.9 eV, respectively (Figure 5e). These binding energy positions are comparable to bulk NaBH_4_, for which B−O and B−H were observed at 192.1 and 187.7 eV, respectively (Figure S14). It is worth mentioning that for NaBH_4_‐TDA@Ni, a slightly higher B−O peak intensity compared to B−H in the B 1s spectra could be due to the carboxylic acid group from TDA consistent with FTIR analysis (Figures 4b and 5e). The presence of surface oxide is often detrimental to the hydrogen materials; however, the XRD and FTIR analyses (Figure 4) showed that these phases are not dominant. For all these materials, the presence of B−O is believed to be the result of partial surface oxidation while transferring the materials to the XPS instrument.

The presence of Ni in the core–shell NaBH_4_@Ni was further confirmed from their respective Ni 2p binding energy positions (Figure 5b, d, f). For NaBH_4_‐TBAB@Ni, the peaks corresponding to Ni 2p_1/2_ and Ni 2p_3/2_ occurred at 868.7 and 851.3 eV, respectively, suggesting the presence of Ni metallic.^[31]^ For NaBH_4_‐ODA@Ni, the Ni 2p_1/2_ and Ni 2p_3/2_ peaks shifted toward higher binding energy values of 868.9 and 851.7 eV, respectively (Figure 5). While in the case of NaBH_4_‐TDA@Ni, the Ni 2p_1/2_ and Ni 2p_3/2_ peaks shifted to even higher binding energy positions at 869.1 and 851.9 eV, respectively (Figure 5b, d, f). This shift toward higher binding energy positions may be because of the differences in the size of surface Ni particles in NaBH_4_‐ODA@Ni and NaBH_4_‐TDA@Ni compared to NaBH_4_‐TBAB@Ni as suggested by the surface core‐level shift model.^[32]^ The additional peak at 853.2 eV in the case of NaBH_4_‐TDA@Ni was ascribed to Ni−O and this may be due to some oxidation of Ni metallic by traces of carboxylate (−COOH) in the material (Figure 5f).^[33]^

It should also be considered that for all the core–shell nanoarchitectures, Ni 2p and B 1s peak positions are very close to the reported values for Ni_*x*_B_*y*_.^[34]^ To further confirm the existence of Ni_*x*_B_*y*_ and identify the oxidation state of Ni in NaBH_4_@Ni, the difference in the binding energies (Δ*E*) between Ni 2p_3/2_ and the first satellite peak can be a good criterion.^[34b]^ The Δ*E* of NaBH_4_‐TBAB@Ni, NaBH_4_‐ODA@Ni, and NaBH_4_‐TDA@Ni was estimated to be 3.4, 3.5, and 3.5 eV, respectively (Figure 5d, f, h). These values are intermediate between the values observed for Ni metal (Δ*E*≈5.8 eV) and Ni_2_B (Δ*E*≈3.2 eV) and cannot be related to NiO (Δ*E*≈1.8 eV).^[34b]^ Hence, the surface of NaBH_4_@Ni nanoarchitectures may be composed of both Ni^0^ and Ni_*x*_B_*y*_.

**Figure 6:**
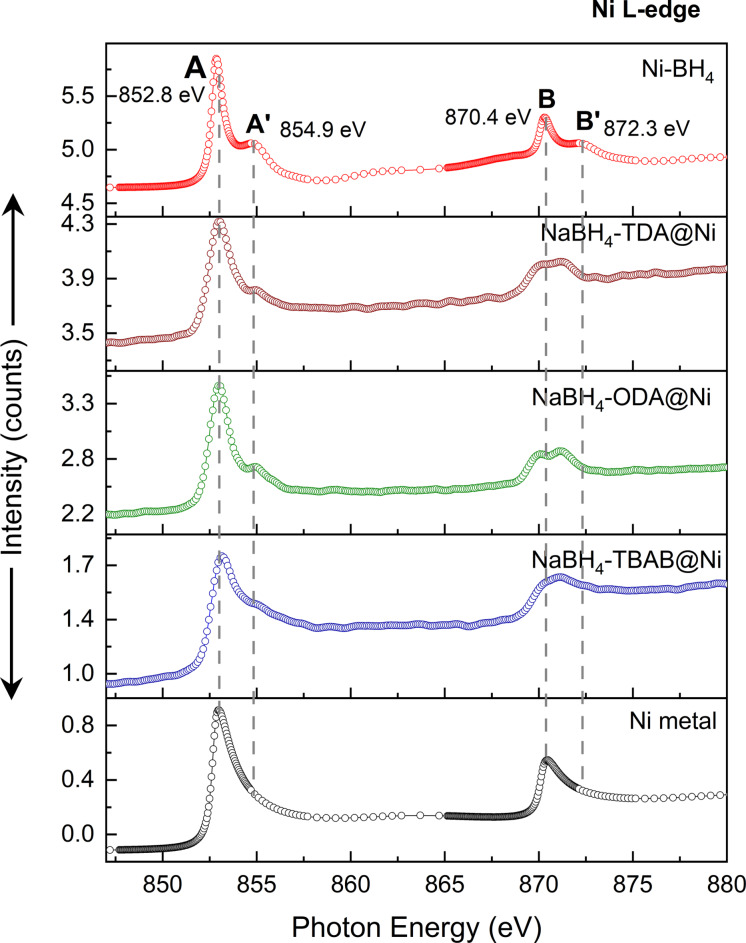
NEXAFS spectra for NaBH_4_@Ni nanostructures as compared to Ni metallic and Ni‐BH_4_ (Ni‐substituted NaBH_4_). The two transitions 2p_3/2_→3d (L_3_‐edge) and 2p_1/2_→3d (L_2_‐edge) are shown as A and B and the shoulder peaks as A′ and B′, respectively. The spectra of Ni‐BH_4_ and nickel borides (NiB, Ni_2_B) were simulated to mimic the Ni atoms in the interface between the Ni shell and NaBH_4_ core (Figure S18).

**Figure 7:**
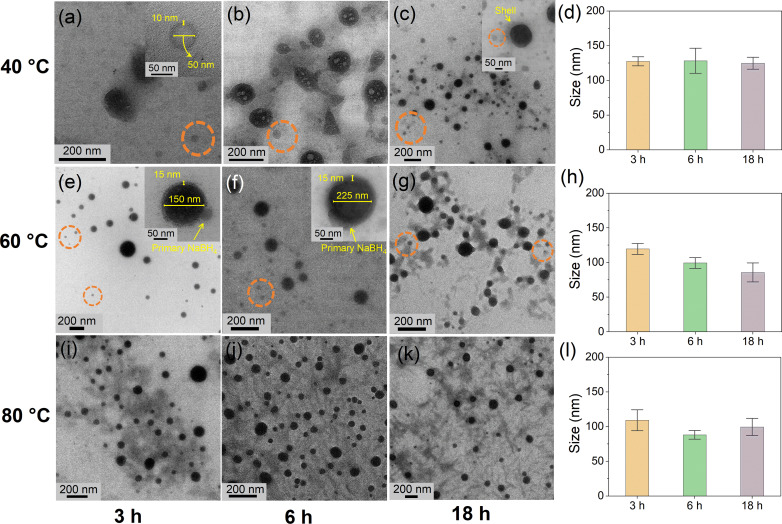
Representative TEM images of the core–shell NaBH_4_‐TBAB@Ni at 40, 60, and 80 °C for (a, e, i) 3 h, (b, f, j) 6 h, and (c, g, k) 18 h, with the corresponding particle sizes in (d,h,l), respectively. The primary NaBH_4_ particles are circled in orange. The inset of (a) shows a remnant (i.e., shell of size 10 nm) of a representative core–shell particle obtained after a prolonged exposure under the electron beam. The inset of (c) shows the core–shell (i.e., inhomogeneous shell) and primary uncoated NaBH_4_ particles. At 60 °C, the insets of (e) and (f) show the representative core–shell particles. The attachment of the primary NaBH_4_ particles with the growing core–shell particles is also identified in the insets of (e) and (f).

Analyses by NEXAFS were further conducted to validate the surface composition and local structure of the NaBH_4_@Ni nanoarchitectures (Figure 6). The NEXAFS analysis revealed that the spectrum of NaBH_4_‐TBAB@Ni resembles a typical Ni metallic, and the broad peak observed at 852.8 eV may be due to the multiplet effects of a more complex local environment near Ni atoms.^[35]^ In comparison, the spectra of NaBH_4_‐ODA@Ni and NaBH_4_‐TDA@Ni showed peaks at 852.8 and 870.4 eV, suggesting the presence of Ni metallic (Figure 6). However, the shoulder peaks (A′ and B′) observed for NaBH_4_‐ODA@Ni and NaBH_4_‐TDA@Ni suggest that their local structures closely resemble the Ni‐BH_4_ phase (Figure 6). The appearance of the shoulder peaks in NaBH_4_‐ODA@Ni and NaBH_4_‐TDA@Ni could be attributed to a less uniform shell on the surface of these materials compared to NaBH_4_‐TBAB@Ni consistent with the TEM observations (Figure 7a). In other words, these results suggest that for NaBH_4_‐ODA@Ni and NaBH_4_‐TDA@Ni, more Ni atoms are located at the interfaces or near the borohydride core than on the surface of the borohydride particle. In addition, the increased intensities in the spectra observed for both NaBH_4_‐ODA@Ni and NaBH_4_‐TDA@Ni compared to NaBH_4_‐TBAB@Ni may indicate an altered electronic state of Ni due to the existence of more electronegative B atoms in the surrounding local structure (Figure 6).^[36]^ Notably, upon close examination of the simulated and experimental NEXAFS spectra (Figures 6 and S16), the Ni^0^/Ni_*x*_B_*y*_ ratio in NaBH_4_@Ni follows a trend: NaBH_4_‐TBAB@Ni>NaBH_4_‐TDA@Ni>NaBH_4_‐ODA@Ni, which is expected to alter the B−H bonds in these materials, and therefore, the decomposition pathways upon hydrogen release.

### Effect of temperature and time on the growth of Ni shell

Our preliminary attempts to synthesize NaBH_4_‐TBAB@Ni at RT produced core–shell particles with a non‐uniform Ni shell, and this was attributed to the slow diffusion of the surfactant molecules (TBAB) away from the NaBH_4_ surface (Figure S19). To further validate this hypothesis, toluene (with a TBAB of solubility <1 mg mL^−1^ at 30 °C) was replaced with cyclohexane. In cyclohexane, where TBAB is not soluble, the formation of an inhomogeneous Ni shell (island growth) over NaBH_4_ particles was observed (Figure S20). This observation shows that the uniformity of the Ni shell can be better controlled by tuning the conditions for the deposition and diffusion of Ni atoms on the surface of the NaBH_4_ particles. For example, by changing the synthetic parameters such as the temperature and reaction time, the growth process can be shifted from the kinetic to thermodynamic control mode.^[37]^ In other words, by effectively balancing the deposition and diffusion processes of Ni atoms on the NaBH_4_ particles, the island growth at low temperatures could be transformed to an epitaxial growth mode at high temperatures, akin to the growth mode regimes of metal‐based core–shell nanoarchitectures.[^37^, ^38^]

For NaBH_4_‐TBAB@Ni, when the synthesis was performed at 40 °C, only a few core–shell NaBH_4_‐TBAB@Ni particles [core (120 nm)/shell (10–15 nm)] were observed after 3 h (Figure 7a), and more core–shell particles appeared after 6–18 h of reaction (Figure 7b, c). During 3–18 h, the progressive disappearance of the primary NaBH_4_ particles (circled in orange) indicates that they are the precursors of the growing core–shell particles, and this is also consistent with the observations on the nucleation and growth processes during Ni‐OAm injections (Figure 1a–c). Moreover, the average size of the core–shell particles remained around 130±5 nm, indicating a negligible growth over time (Figure 7d). It should be noted that the size discrepancy between the core–shell particles shown in Figure 7a and the average particle size distribution in Figure 7d is because of the presence of several particles of size <100 nm (termed as “primary” core–shell NaBH_4_@Ni particles, Figure S21 and Video S1). A typical primary NaBH_4_@Ni particle [core (50 nm)/shell (10 nm)] after the decomposition under the electron beam is shown in Figure 7a, (inset, circled orange, and Video S2). Overall, an inhomogeneous Ni shell was observed even after 18 h at 40 °C (Figure 7a–d).

When the temperature was increased to 60 °C, core–shell NaBH_4_‐TBAB@Ni particles of mean size around 130±5 nm were obtained after 3 h (Figure 7e, f). After 6–18 h, many core–shell particles of core size around 110–80±5 nm and shell thickness around 15±2 nm were obtained (Figure 7f, g). As compared to 40 °C, we believe that the diffusion rate of Ni atoms on the NaBH_4_ surface is greater than their deposition rate at 60 °C, which may have resulted in a homogeneous/smoother Ni shell.^[37]^ It should be noted that after 18 h the mean particle size was around 80 nm primarily because of the emergence of many smaller core–shell particles (Figure 7h). The emergence of these core–shell particles could be due to the “reconstruction” and/or “digestive ripening”, where smaller particles grow at the expense of bigger particles until a dynamic equilibrium is established.^[39]^ However, the exact growth mechanism remains unclear.

At 80 °C, the fast nucleation resulted in the formation of the core–shell NaBH_4_‐TBAB@Ni particles of size around 100±5 nm along with isolated Ni^0^ nanoparticles (Figures 7i–k and S22). In this case, the faster collision and reduction processes between NaBH_4_ and Ni‐OAm may have favored the homogeneous nucleation of isolated Ni^0^ particles in solution. Overall, after 6–18 h, the particle size remained around 95–100±5 nm (Figure 7j–l). The effect of temperature and time on the cube‐like NaBH_4_‐ODA@Ni and bar‐like NaBH_4_‐TDA@Ni nanoarchitectures has also been investigated and similar observations were made (Figures S23–S28).

**Scheme 1:**
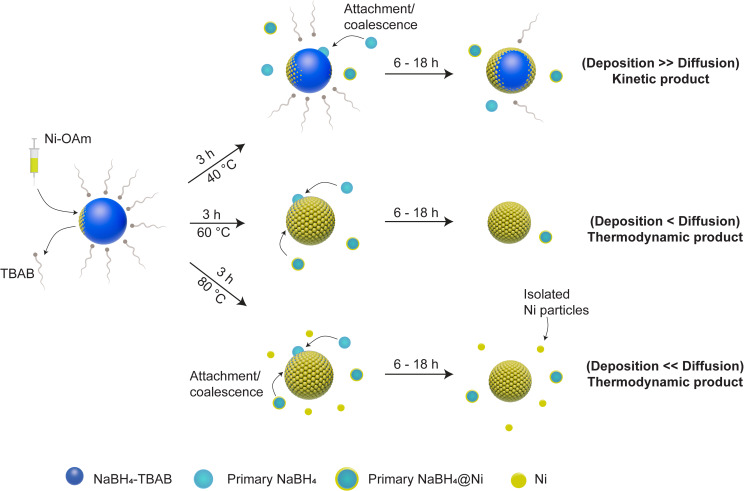
Illustration of the nucleation and growth processes of NaBH_4_‐TBAB@Ni at different temperatures. For the sake of simplicity, the Ni_*x*_B_*y*_ phase is not shown. The size of the particles is based on the average size of core–shell particles shown in Figure 7d, h, l.

Based on the above, a growth model for the formation of the core–shell NaBH_4_‐TBAB@Ni is proposed and illustrated in Scheme 1. During Ni‐OAm injections, the excess of TBAB molecules would diffuse away from the surface of the NaBH_4_ particles, where the Ni^II^ ions are deposited on the exposed facets of the NaBH_4_ particles before their reduction to Ni^0^. During the period (3, 6, or 18 h) at a reaction temperature (40, 60, or 80 °C), the Ni shell would grow on the surface of the NaBH_4_ particles and Ni^0^ atoms diffuse into the subsurface of NaBH_4_ (Scheme 1). At 40 °C, after 3 h, the primary NaBH_4_ and NaBH_4_@Ni particles coalesce with the growing core–shell NaBH_4_‐TBAB@Ni particles, and this combined with the low diffusion and high deposition rates of Ni atoms on the NaBH_4_ surfaces would lead to inhomogeneous Ni shell (kinetic product). In contrast, at 60 °C, the higher diffusion rate of Ni atoms on the NaBH_4_ surface compared to their deposition rate would lead to homogeneously coated NaBH_4_‐TBAB@Ni (thermodynamic product). At a higher synthesis temperature of 80 °C, the sphere‐like core–shell NaBH_4_‐TBAB@Ni particles with homogeneous Ni shells would rapidly form; however, in this case, isolated Ni^0^ particles are also obtained owing to the higher diffusion rates and collisions with the Ni atoms in solution (Scheme 1).

**Scheme 2:**
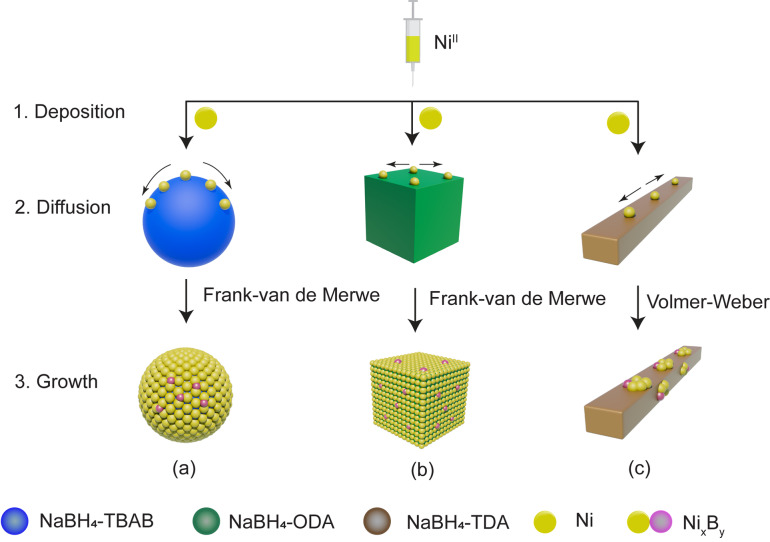
Different growth processes are illustrated for (a) NaBH_4_‐TBAB@Ni, (b) NaBH_4_‐ODA@Ni, and (c) NaBH_4_‐TDA@Ni nanoarchitectures. The growth mechanism involves the deposition and reduction of Ni^II^ on the surface of NaBH_4_ (indicated **1** and **2**), the Ni^0^ atoms diffuse further onto the surface of NaBH_4_ and form Ni_*x*_B_*y*_ in NaBH_4_ (indicated **2** and **3**), and finally the growth occurs via concomitant deposition and diffusion processes (indicated **3**).

Here, it should be emphasized that the growth mode can be affected by the surfaces and interfaces of NaBH_4_ and Ni.^[40]^ Surface energy values for NaBH_4_ (β phase) were reported as 1.89, 1.75, and 1.36 J m^−2^ for the planes (001), (110), and (100), respectively.^[41]^ In contrast, the surface energies of the Ni (fcc) were reported to be 2.43, 2.37, and 2.01 J m^−2^ for (100), (110), and (111), respectively.^[42]^ The surface energies of Ni are larger than NaBH_4_; therefore, the thermodynamic conditions suggest that a layered growth (Frank—van de Merwe) mode would not be favored.^[40]^ More concisely, high surface energy would promote an island growth (Volmer–Weber) mode of Ni over NaBH_4_ particle (Scheme 2). In the present work, however, we believe that the surface of NaBH_4_ is pre‐conditioned by the presence of surface ligands (TBAB, ODA, or TDA) and these could minimize the surface energy barriers while favoring the growth of the Ni shell over NaBH_4_ as previously observed for the Pd/Ag systems.^[43]^

Considering the above findings, TBAB and ODA ligands seemed to favor a conformal growth of the Ni shell over NaBH_4_, while TDA ligand promoted an island growth of Ni. The conformal epitaxial growth concerning NaBH_4_‐TBAB@Ni and NaBH_4_‐ODA@Ni can also be because of the smaller particle sizes (≈80–150 nm) and (≈300 nm), respectively, compared to NaBH_4_‐TDA@Ni (≈900 nm). In this case, Ni atoms would likely take much less time to diffuse and “stabilize” on the surface of smaller NaBH_4_ particles because of the shorter diffusion distances (Scheme 2).^[44]^

### Correlating the structure and hydrogen release of NaBH_4_@Ni nanoarchitectures

**Figure 8:**
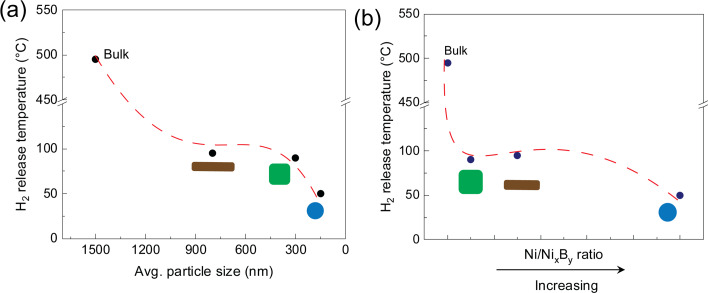
Correlation of (a) particle size and (b) Ni/Ni_*x*_B_*y*_ ratio with the H_2_ release for sphere‐like NaBH_4_‐TBAB@Ni, cube‐like NaBH_4_‐ODA@Ni, and bar‐like NaBH_4_‐TDA@Ni. The Ni/Ni_*x*_B_*y*_ ratio in (b) was estimated from the NEXAFS spectra (Figure 6).

The hydrogen release profiles from the core–shell nanoarchitectures were obtained by using thermogravimetric analysis (TGA)/differential scanning calorimetry (DSC) coupled with mass spectrometry (MS) (Figure S29). As expected, owing to the nanosizing and the presence of the Ni^0^/Ni_*x*_B_*y*_ species, all the NaBH_4_@Ni nanoarchitectures showed hydrogen release at lower temperatures compared to bulk NaBH_4_ (>500 °C, Figure 8). For instance, NaBH_4_‐TBAB@Ni, NaBH_4_‐ODA@Ni, and NaBH_4_‐TDA@Ni started to release pure hydrogen at around 50, 90, and 95 °C (Figure 8), while the major hydrogen release for these materials peaked between around 465–508, 465–512, and 300–480 °C, respectively (Figure S29). The hydrogen release temperatures also corroborated well with the mass losses observed from the TGA curves (Figure S29a, c, e). The mass loss for NaBH_4_‐TBAB@Ni, NaBH_4_‐ODA@Ni, and NaBH_4_‐TDA@Ni was around 2.5, 5.4, and 24 mass% until 500 °C, respectively (Figure S29). A higher mass loss in the case of NaBH_4_‐TDA@Ni could be because of Na evaporation along with H_2_ release consistent with the island growth of Ni (Figures S26a and S29e).

Previously, we demonstrated that the melting temperature and hydrogen release could be tuned by tailoring the size and shapes of NaBH_4_ such as spheres, cubes, and/or bars.^[23]^ For example, NaBH_4_‐TBAB (spheres), NaBH_4_‐ODA (cubes), and NaBH_4_‐TDA (bars) started to release a significant amount of hydrogen at around 200, 400, and 100 °C, respectively. In the present work, NaBH_4_‐TBAB@Ni, NaBH_4_‐ODA@Ni, and NaBH_4_‐TDA@Ni started to release hydrogen as low as around 50, 90, and 95 °C, respectively (Figures 8 and S29). The improved hydrogen release from the core–shell NaBH_4_@Ni materials may be due to the destabilization induced by the Ni species (Ni^0^ or Ni_*x*_B_*y*_) consistent with the NEXAFS analysis. The improved hydrogen release could also be due to the particle size, shape effects, and/or the core–shell structure. For example, the sphere‐like NaBH_4_‐TBAB@Ni showed the lowest temperature for hydrogen release, which could be due to its small particle size compared to the cube‐like NaBH_4_‐ODA@Ni and bar‐like NaBH_4_‐TDA@Ni nanoarchitectures (Figures 6 and 8b). Figure 8b depicts a correlation between the increasing Ni^0^/Ni_*x*_B_*y*_ ratio and hydrogen release of NaBH_4_‐TBAB@Ni, NaBH_4_‐ODA@Ni, and NaBH_4_‐TDA@Ni. These results reveal that improved hydrogen release may be because of varying content of the Ni^0^/Ni_*x*_B_*y*_ species. Metal borides have widely been identified as effective catalysts in improving the hydrogen release properties of various borohydrides.^[45]^ These studies also showed that metal borides exhibit long‐range disordered structures with several vacant electron orbitals and unsaturated coordination sites. In the present study, the presence of the Ni_*x*_B_*y*_ species in NaBH_4_@Ni is expected to accelerate electron loss from BH_4_^−^ anions and H‐atoms, which combine to release H_2_. In the presence of the Ni_*x*_B_*y*_ species, the unoccupied B orbital and lone electrons in N would establish the donor–acceptor pairs which may also promote the extraction of H from −NH_2_ and release H_2_.^[46]^ The effect of the remaining surfactants may also result in the improved H_2_ release through H^*δ*+^/H^*δ*−^ interactions (e.g., in NaBH_4_‐ODA@Ni and NaBH_4_‐TBAB@Ni).

These results show that the NaBH_4_@Ni nanoarchitectures can be effectively destabilized by modifying the ratio of in‐situ formed Ni^0^ and/or Ni_*x*_B_*y*_ species toward efficient hydrogen release. Further control of the confinement of the destabilized borohydride cores via Ni^0^ or Ni_*x*_B_*y*_ species is expected to improve the reversibility of NaBH_4_@Ni, especially, the formation of boron phases; as Ni_*x*_B_*y*_ inside the Ni shell could provide a path toward limiting the boron loss and increasing the hydrogen uptake in borohydrides.^[47]^ The present strategy can also be extended to other core–shell complex hydrides that could provide practical solutions to meet the hydrogen storage targets.

## Conclusions

A simple strategy for controlling the core–shell nanoarchitectures and hydrogen properties of NaBH_4_ is reported. The sphere, cube, and bar‐like NaBH_4_ nanoarchitectures were synthesized by using different surfactants and coated with Ni to stabilize the NaBH_4_ core as well as suppress its melting and Na evaporation during hydrogen release/uptake. All the NaBH_4_@Ni nanoarchitectures showed improved hydrogen release compared to bulk NaBH_4_. The hydrogen release occurred at around 50, 90, and 95 °C for the sphere‐like, cube‐like, and bar‐like core–shell nanoarchitectures, respectively, compared to bulk NaBH_4_, which released hydrogen above 500 °C. In particular, the onset temperature for hydrogen release of the NaBH_4_@Ni nanoarchitectures varied depending on the particle size, Ni shell, and the presence of the Ni^0^/Ni_*x*_B_*y*_ species. Structural investigations revealed that the formation of a uniform Ni shell (metallic) can help suppress the evaporation of Na and trigger hydrogen release at lower temperatures. Proper substitution of Ni in the NaBH_4_ core and the formation of the Ni_*x*_B_*y*_ species could better destabilize the borohydride core. We believe that this study can serve as a guideline to further tune the hydrogen properties of transition metal‐coated borohydrides.

## Experimental Section

### Materials and methods

All the experiments were performed under an inert atmosphere in an argon‐filled LC‐Technology glovebox (O_2_ and H_2_O<1 ppm). Sodium borohydride (NaBH_4_, 99.99 %, trace metals basis), nickel chloride hexahydrate (NiCl_2_ ⋅ 6H_2_O, 99 %) isopropylamine (IPA, 99.5 %), anhydrous dimethyl sulfoxide (DMSO), anhydrous toluene, cyclohexane, octadecylamine (ODA, 99 %), tetrabutylammonium bromide (TBAB, 99 %) dried at 100 °C for at least 6 h, tridecanoic acid (TDA, 99 %), and oleylamine (>97 %) were all purchased from Sigma‐Aldrich.

### Synthesis of NaBH_4_ nanoarchitectures

To a solution of NaBH_4_ (100 mm in IPA), a set concentration of a surfactant stock solution (200 mm in IPA) was added while stirring the mixture at 500 rpm and RT. After stirring for 1 h, the homogenized mixture was dried at 2 mbar, 30 °C.

### Preparation of Ni‐oleylamine complex

In a typical phase transfer experiment, about 13 mg of anhydrous NiCl_2_ was dissolved in 5 mL DMSO at 70 °C under stirring for 2 h and cooled down to RT. The color of the solution became yellow/pale green, which is the characteristic of Ni‐DMSO complex formation according to Equation (1).^[48]^ In a separate vessel, oleylamine (≈150 mg) was dissolved in 5 mL toluene and added at once to the Ni‐DMSO solution while stirring. The ligand exchange between DMSO and OAm was performed under vigorous stirring for 5 min. At this stage, the color of the solution becomes light green due to the formation of the Ni‐OAm complex [Eq. (2)]. Then, 5 mL cyclohexane was added to the mixture and stirred for 5 min. The mixture on stirring was stopped and Ni‐OAm at once transferred to the upper phase of cyclohexane/toluene (Scheme S1). The solution was placed undisturbed for a few hours, and the upper phase was collected and analyzed. The bottom phase upon addition of a stronger ligand than amine (i.e., ethylenediamine) did not give any evidence of Ni‐ethylenediamine complex formation. Thus, Ni‐OAm was successfully prepared and transferred to the upper phase. The concentration measurements of the upper and lower phases by inductively coupled plasma mass spectrometry (ICP‐MS) showed that phase transfer efficiency was 85 %.

(1) $$\left[\mathrm{Ni}\left(\mathrm{DMSO}\right){}_{6}\right]{}^{2+}+y\;\mathrm{Cl}{}^{-}\left(\mathrm{solv}\right)\vec{\leftarrow }\left[\mathrm{Ni}\left(\mathrm{DMSO}\right){}_{x}\mathrm{Cl}{}_{y}\right]{}^{2-y}+(6-x)\mathrm{DMSO}$$

(2) $$\left[\mathrm{Ni}\left(\mathrm{DMSO}\right){}_{x}\mathrm{Cl}{}_{y}\right]{}^{2-y}+6\;\mathrm{OAm}\to \left[\mathrm{Ni}\left(\mathrm{OAm}\right){}_{x}\right]{}^{2+}\mathrm{Cl}{}_{y}+x\;\mathrm{DMSO}$$

### Synthesis of NaBH_4_@Ni nanoarchitectures

5 mg mL^−1^ of the surfactant stabilized NaBH_4_ was dispersed in 5 mL of toluene and sonicated to get a homogeneous suspension. Nickel precursor Ni‐OAm was separately prepared in toluene to get a final concentration of 0.25, 0.5, 1, 3, 5, and/or 10 mm (for 10 mL). The nickel precursor was injected at a rate of 100 μL min^−1^ into the NaBH_4_‐surfactant suspension (with and without trioctylphosphine ligand, TOP), 250 rpm at 25, 40, 60, and/or 80 °C for (i) 15, 30, and 45 min, and (ii) 1, 3, 6, and 18 h.

### Reduction kinetics

5 mg mL^−1^ of the TBAB‐stabilized NaBH_4_ was dispersed in toluene and sonicated for a few seconds. The precursor Ni‐OAm (final concentration 0.5 mm) was added to the NaBH_4_ solution under stirring at 500 rpm and RT. The sample aliquot (100 μL) was taken at regular intervals and added to 500 μL of H_2_O to hydrolyze/decompose NaBH_4_. The decomposed products were removed via centrifugation at 14000 rpm, 10 min, and RT. The supernatant was further mixed with 100 μL of absolute ethanol (to help in homogenizing and combustion of the organic matter) and 300 μL of concentrated nitric acid. The mixture was baked at 90 °C for at least 2 h, appropriately diluted, and the concentration of Ni^II^ was analyzed by ICP‐MS.

## Characterizations

**Electron microscopy**: Microscopic analysis was done by TEM on Philips CM 200 (Eindhoven, the Netherlands) operated at 200 kV. HAADF‐STEM images were taken by field emission gun (FEG)‐scanning TEM (FEG‐STEM, JEOL, JEM‐F200 Multipurpose FEG‐STEM, Tokyo, Japan) at 200 kV at a camera length of around 120 mm and an angle of ±15°. The materials were dispersed in toluene followed by short ultrasonication and then dropped onto a carbon‐coated copper grid. The samples were enclosed in an argon‐filled bottle to minimize air exposure and then transferred to the TEM facility.

**ICP‐MS**: The core–shell materials were dissolved in concentrated nitric acid and baked at 90 °C for at least 2 h. The solution was appropriately diluted, and the Ni content was measured.

**XRD**: Crystalline phases were determined by XRD on the X'pert Multipurpose XRD system operated at 40 mA and 45 kV with a monochromated Cu K_α_ radiation (*λ*=1.541 Å) from 10 to 80°. The materials were protected against oxidation in the air by a Kapton foil.

**FTIR spectroscopy**: FTIR spectroscopy was conducted on a Bruker Vertex 70 V. The materials were mixed with KBr and loaded in an air‐tight chamber fitted on a Harrick‐Scientific Praying Mantis Diffuse Reflectance Infrared Fourier Transform Spectroscopy (DRIFTS) accessory. The spectra were collected at RT from 600 to 3000 cm^−1^ over 124 scans with a resolution of 1 cm^−1^.

**XPS**: The chemical properties of the surface of the nanomaterials were characterized by XPS using a Thermo Scientific ESCALAB250Xi, UK spectrometer (base pressure below 2×10^−6^ Pa). Powder materials were pressed on high‐quality indium substrates, placed in a container filled with argon and transferred to the spectrometer to minimize exposure to air. The XPS spectra were collected using a monochromatic Al K_α_ (1486.7 keV) X‐ray source at 150 W power. Survey scans were collected at 100 eV pass energy with an energy step of 0.5 eV, while high‐resolution spectra were acquired at the 20 eV pass energy and 0.1 eV energy step. The data were analyzed and processed using Advantage and CasaXPS.

**NEXAFS**: The NEXAFS measurements were performed at the B K‐edge, Na K‐edge, and Ni L_2_ and L_3_ edge using the Soft X‐rays (SXR) beamline of the Australian Synchrotron. Samples were stored in sealed vials and spread across carbon tape for partial electron yield detection in the glovebox. The prepared samples were stored in a glove bag filled with argon during the transportation from the glovebox to the sample loading dock to avoid contamination. All data processing and analysis were performed in Igor Pro using the QANT NEXAFS tool.^[49]^

**NEXAFS theoretical calculations**: Full multiple scattering with spin‐orbit calculations employing muffin tin approximation was performed using FDMNES code to calculate Ni L_2_‐L_3_ NEXAFS for Ni metal, NiB, and Ni_2_B structures.^[50]^ To simulate the NEXAFS features, the calculation was performed including all atoms within 3.5 Å away from the Ni absorber. To calculate Ni L_2_‐L_3_ NEXAFS for Ni‐substituted NaBH_4_ and Ni(OH)_2_ structures, simulations were performed with the finite difference method (FDM) within full potential up to 3.5 Å.^[51]^ In FDM calculations, Hedin–Lundqvist (HL) exchange‐correlation potentials were used in the self‐consistent calculations, which resulted in a better agreement with the experimental data.

## Conflict of interest

The authors declare no conflict of interest.

1

## Supporting information

As a service to our authors and readers, this journal provides supporting information supplied by the authors. Such materials are peer reviewed and may be re‐organized for online delivery, but are not copy‐edited or typeset. Technical support issues arising from supporting information (other than missing files) should be addressed to the authors.

Supporting Information

## Data Availability

The data that support the findings of this study are available in the Supporting Information.
