# Supplementary material for: Core–shell NaBH4@Ni Nanoarchitectures: A Platform for Tunable Hydrogen Storage
Source: ChemSusChem. 2022 Jul 13;15(17):e202200664. doi: 10.1002/cssc.202200664 (PMC9542058; doi:10.1002/cssc.202200664)
Supplement: Supplementary file 1 — Supporting Information [file CSSC-15-0-s001.pdf]

ChemSusChem

Supporting Information

Core–shell $NaBH_4$@Ni Nanoarchitectures: A Platform for Tunable Hydrogen Storage

Muhammad Saad Salman, Yuwei Yang, Muhammad Zubair, Nicholas M. Bedford, and Kondo-Francois Aguey-Zinsou*© 2022 The University of Sydney. ChemSusChem published by Wiley-VCH GmbH. This is an open access article under the terms of the Creative Commons Attribution License, which permits use, distribution and reproduction in any medium, provided the original work is properly cited.

Table of Contents…

Supporting Schemes........2

1. A schematic for the synthesis of Ni-OAm complex via the phase transfer method.......2

Supporting Figures........3

1. The spectrometric characterization of the Ni complexes........3
2. Effect of TOP on the formation of $NaBH_4$-TBAB@Ni particles........4
3. Line-scan analysis of a core-shell particle........6
4. Core-shell $NaBH_4$-TBAB@Ni particle after decomposition........6
5. $NaBH_4$-TBAB@Ni particles during Ni-OAm injections........7
6. $NaBH_4$-ODA@Ni particles during Ni-OAm injections........8
7. $NaBH_4$-TDA@Ni particles during Ni-OAm injections........9
8. Irregular shapes of bare $NaBH_4$ after Ni coating........10
9. XRD pattern of pristine $NaBH_4$........11
10. FTIR spectra of pristine $NaBH_4$........12
11. XRD intensity comparison of $NaBH_4$-TBAB and $NaBH_4$-TBAB@Ni........13
12. XRD intensity comparison of $NaBH_4$-ODA and $NaBH_4$-ODA@Ni........14
13. XRD intensity comparison of $NaBH_4$-TDA and $NaBH_4$-TDA@Ni........15
14. XPS spectra of $NaBH_4$-TBAB@Ni........16
15. XPS spectra of $NaBH_4$-ODA@Ni........17
16. XPS spectra of $NaBH_4$-TDA@Ni........18
17. XPS spectra of pristine $NaBH_4$........19
18. Simulated NEXAFS spectra........21
19. Island growth of Ni shell at RT........22
20. Core-shell $NaBH_4$@Ni in cyclohexane with discrete Ni particles........23
21. Primary core-shell particles........24
22. Isolated Ni particles with core-shell $NaBH_4$-TBAB@Ni at 80 °C........25
23. Core-shell $NaBH_4$-ODA@Ni at different temperatures and times........26
24. Core-shell $NaBH_4$-TDA@Ni at different temperatures and times........27
25. TGA/DSC and hydrogen release profiles of core-shell nanoarchitectures........30

Supporting Videos........31

1. **Video S1**........31
2. **Video S2**........31

References........32

Supporting Schemes

1. A schematic for the synthesis of Ni-OAm complex via the phase transfer method.

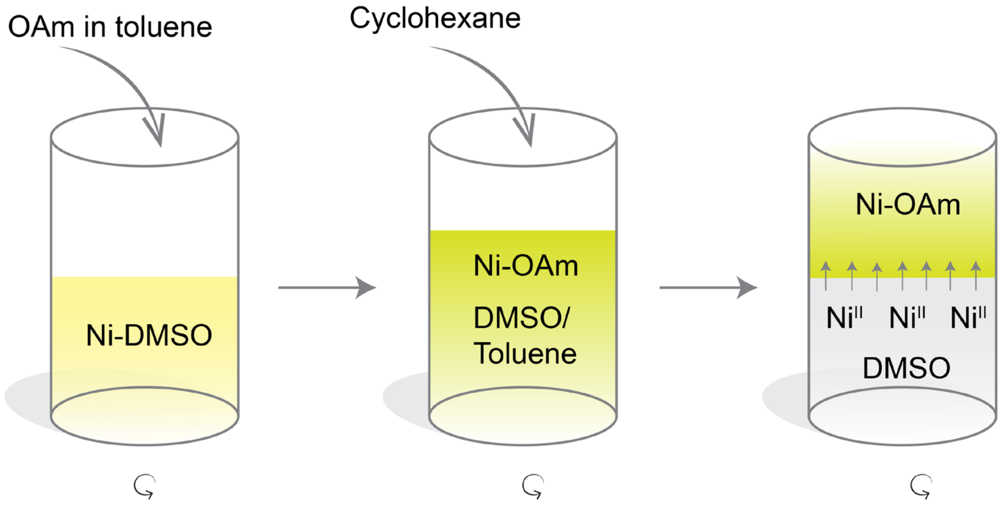

Scheme S1. A schematic illustration for the preparation of Ni-OAm via phase transfer.

Supporting Figures

1. The spectrometric characterization of the Ni complexes

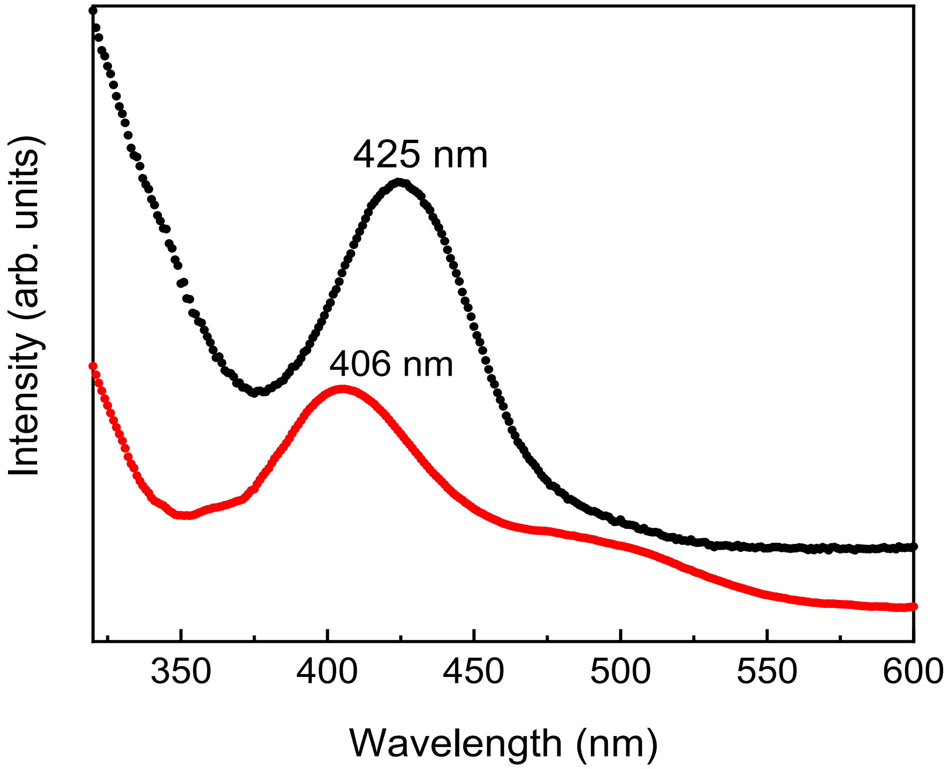

Figure S1. UV-vis of Ni-DMSO complex in DMSO (black) and Ni-OAm complex in toluene (red). The concentration of each complex was 1 mM. The difference in the wavelengths in black and red indicate the formation of different complexes.

2. Effect of TOP on the formation of $NaBH_4$-TBAB@Ni particles

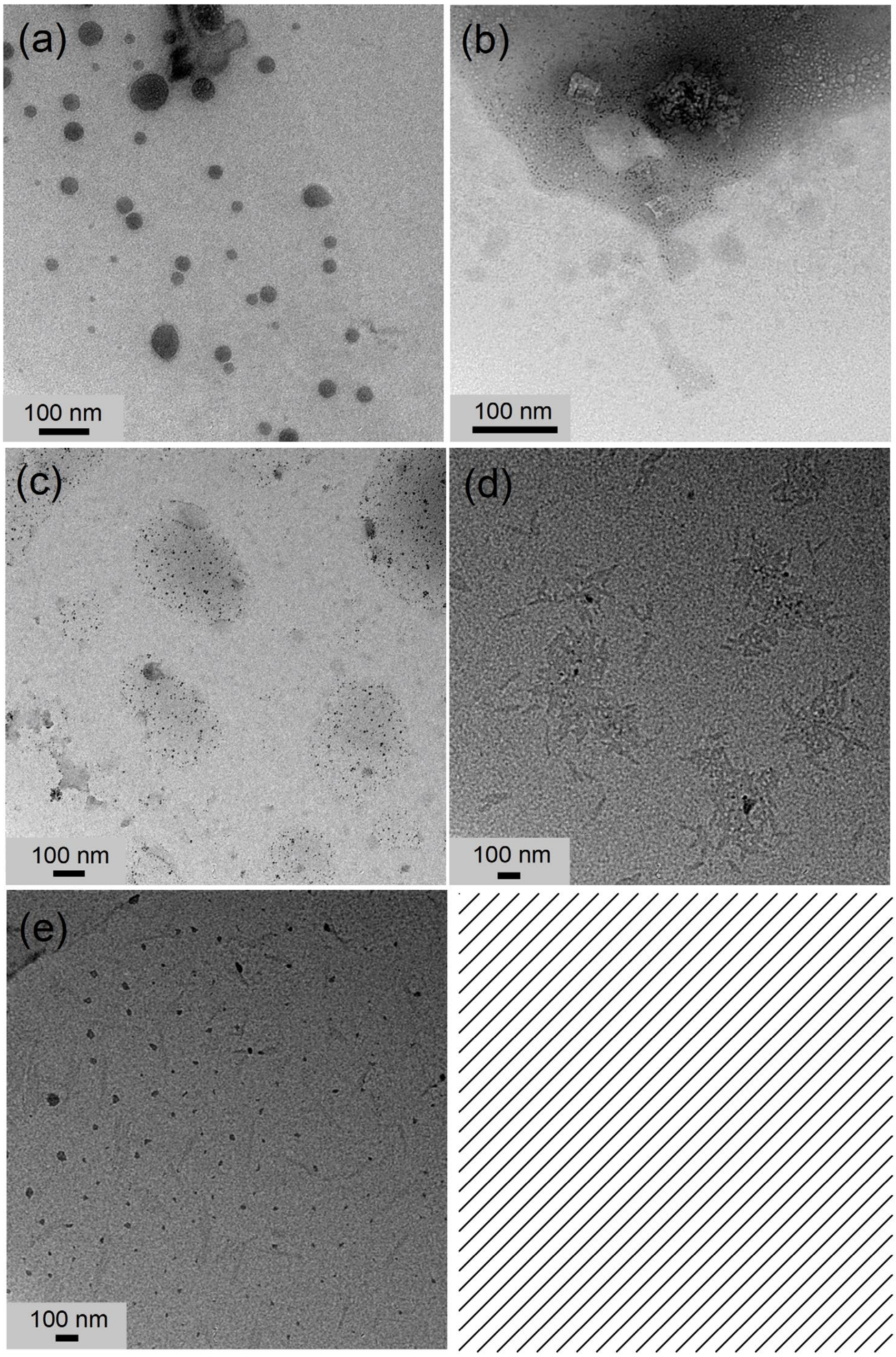

Figure S2. $NaBH_4$-TBAB@Ni obtained at a TOP concentration of (a) 50 uM, (b) 100 uM, (c) 5 mM, (d) 10 mM, and (e) 15 mM. The concentration of $NaBH_4$-TBAB and Ni-OAm were 5 mg mL^{-1} and 0.5 mM, respectively.

Explanation of Figure S2

The addition of TOP was found beneficial for the growth of core-shell particles. It was previously observed that the presence of TOP and OAm could provide better stabilization of Ni particles via Ni-TOP coordination and avoid their agglomeration of Ni particles than OAm alone.[1] We also confirmed this effect during the purification of the core-shell materials where the dark suspension in toluene was very stable with TOP concentrations between 5–15 mM and it was difficult to recover all the suspended particles from the solution (data not shown). We observed that the stable suspension consisted of Ni particles in toluene. A small amount of TOP ligand was sufficient for the stabilization of the Ni particles; however, a high concentration of TOP collapsed the core-shell structure (Figure S2). We noted that with a TOP concentration between 0.1–5 mM (Figure S2b, c), the $NaBH_4$ core was destroyed, and isolated Ni particles were obtained (Figure S2c). Similar results were obtained for 5–15 mM of TOP.

3. Line-scan analysis of a core-shell particle

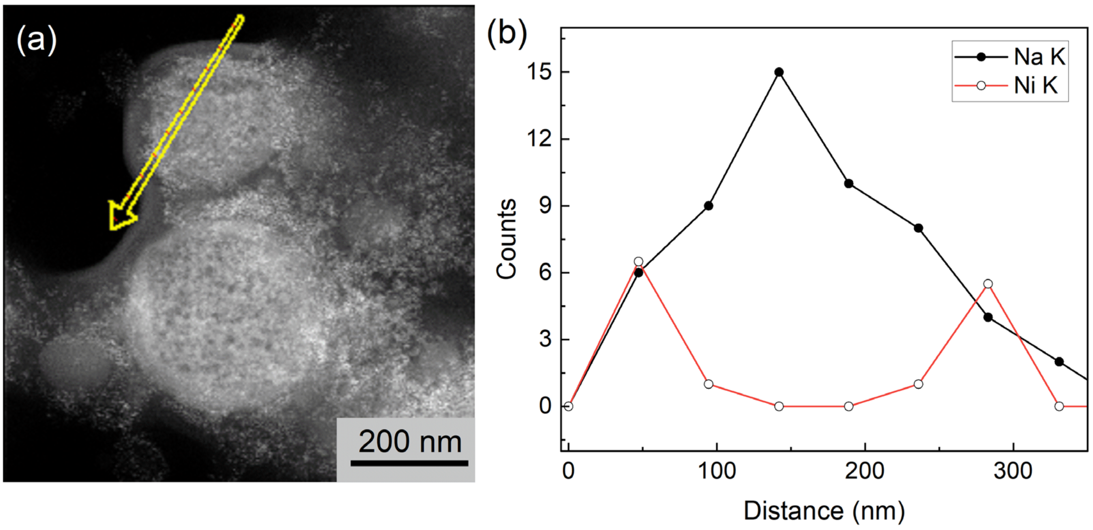

Figure S3. (a) HAADF image of $NaBH_4$-TBAB@Ni and (b) the corresponding line-scan analysis based on the arrow shown in (a). The line-scan analysis (b) shows that Ni is most strongly present on the outer rim (periphery) of a core-shell particle and the concentration of Na increases toward the centre, suggesting the presence of the Na rich core (i.e. $NaBH_4$).

4. Core-shell $NaBH_4$-TBAB@Ni particle after decomposition

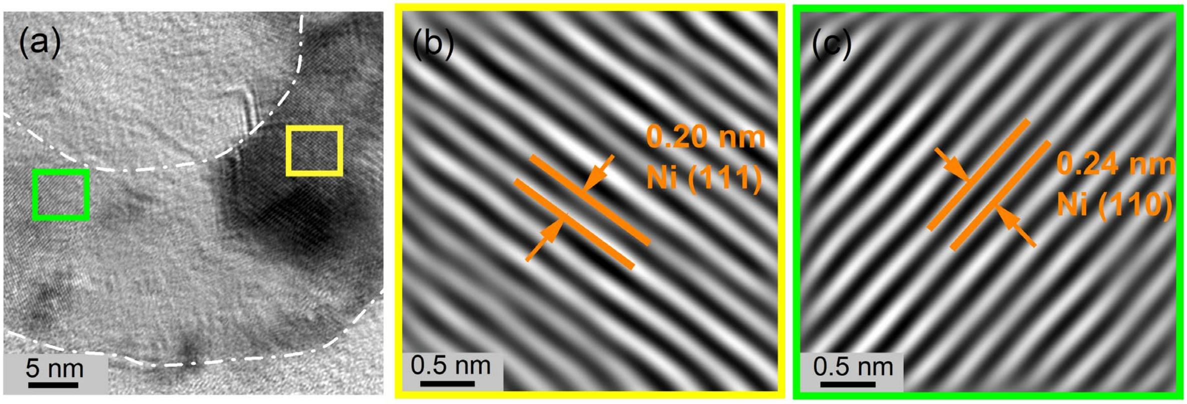

Figure S4. HRTEM image (a) of a representative core-shell $NaBH_4$-TBAB@Ni particle obtained after 18 h at 60 °C. The image was taken by leaving the core-shell particle under the electron beam for a prolonged duration to decompose the $NaBH_4$ core, so the Ni shell is clearly visible. (b) and (c) show the fast-Fourier transform (FFT) from the areas marked in yellow and green in (a), respectively. The d-spacing in (b) and (c) correspond to Ni (111) and Ni (110), respectively.

5. $NaBH_4$-TBAB@Ni particles during Ni-OAm injections

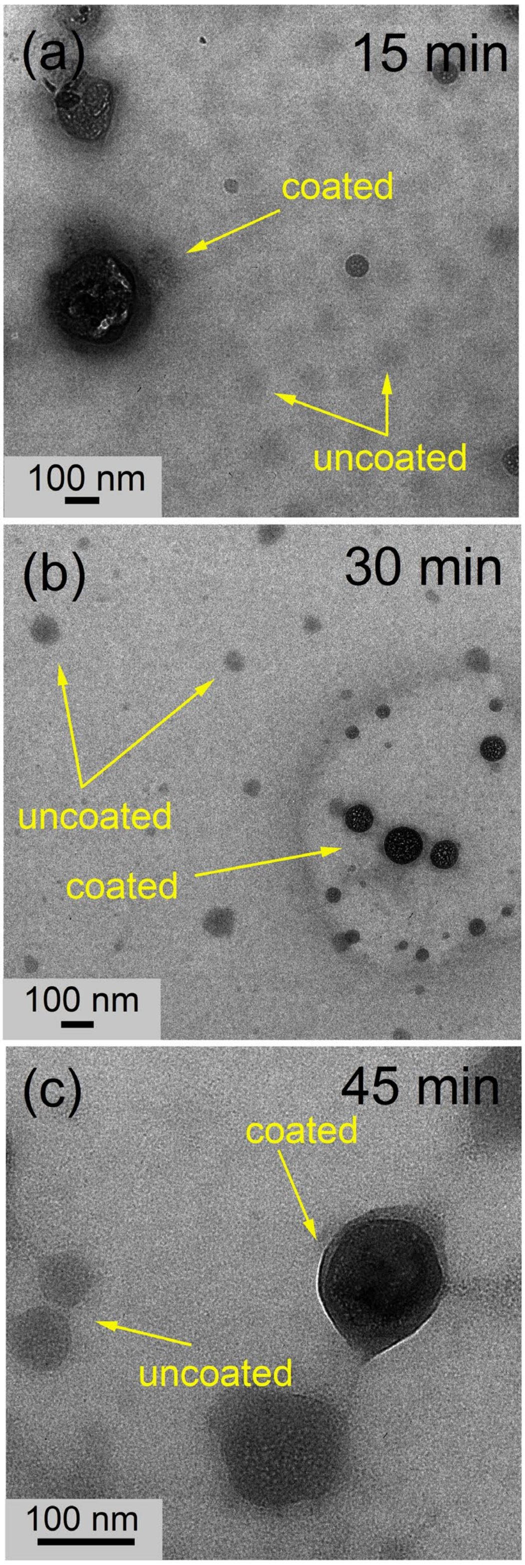

Figure S5. TEM images of $NaBH_4$-TBAB@Ni after (a) 15, (b) 30 and (c) 45 min of Ni-OAm injections at 40 °C.

6. $NaBH_4$-ODA@Ni particles during Ni-OAm injections

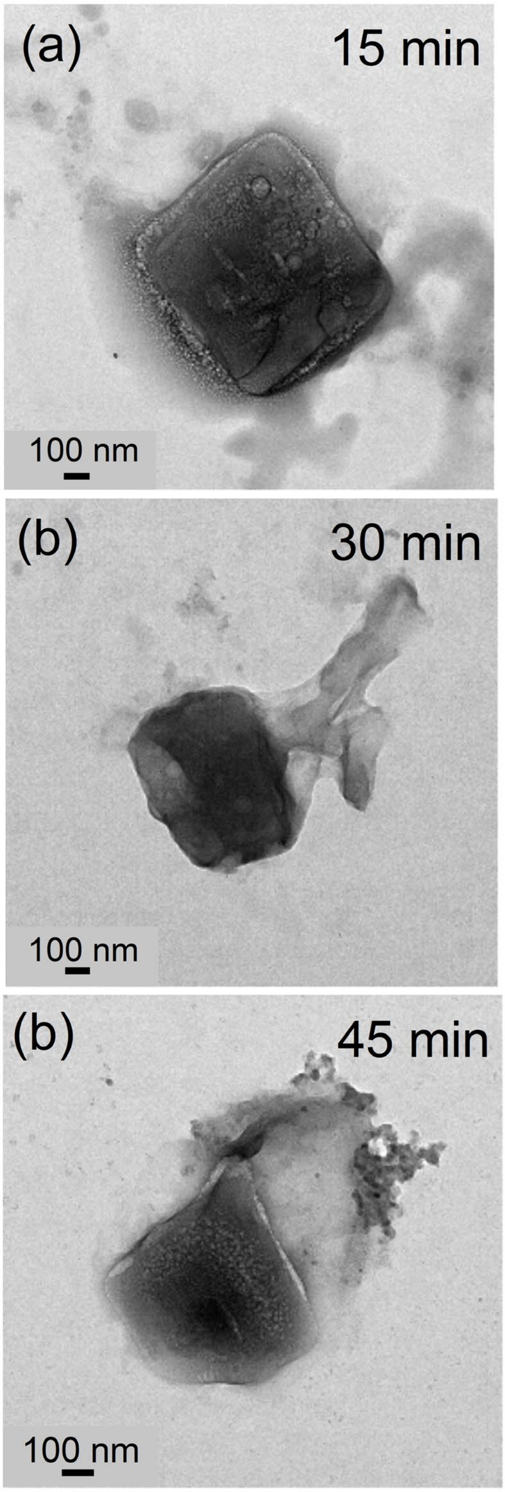

Figure S6. TEM images of $NaBH_4$-ODA@Ni after (a) 15, (b) 30 and (c) 45 min of Ni-OAm injections at 40 °C.

7. $NaBH_4$-TDA@Ni particles during Ni-OAm injections

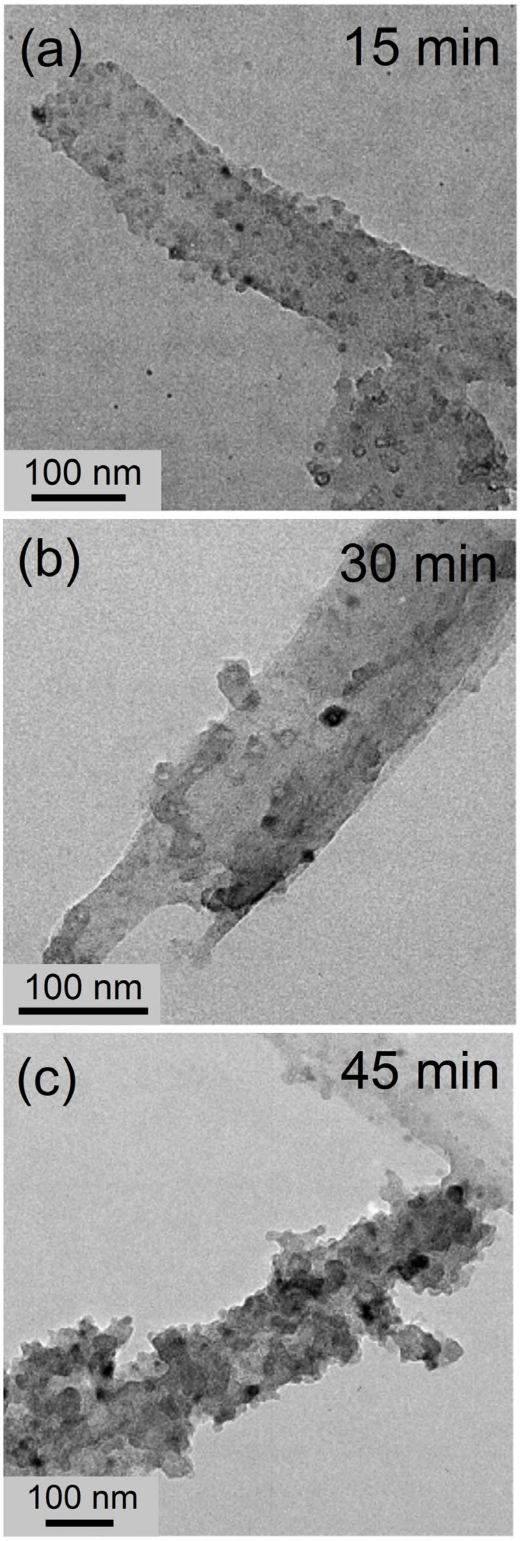

Figure S7. TEM images of $NaBH_4$-TDA@Ni after (a) 15, (b) 30 and (c) 45 min of Ni-OAm injections at 40 °C.

8. Irregular shapes of bare $NaBH_4$ after Ni coating

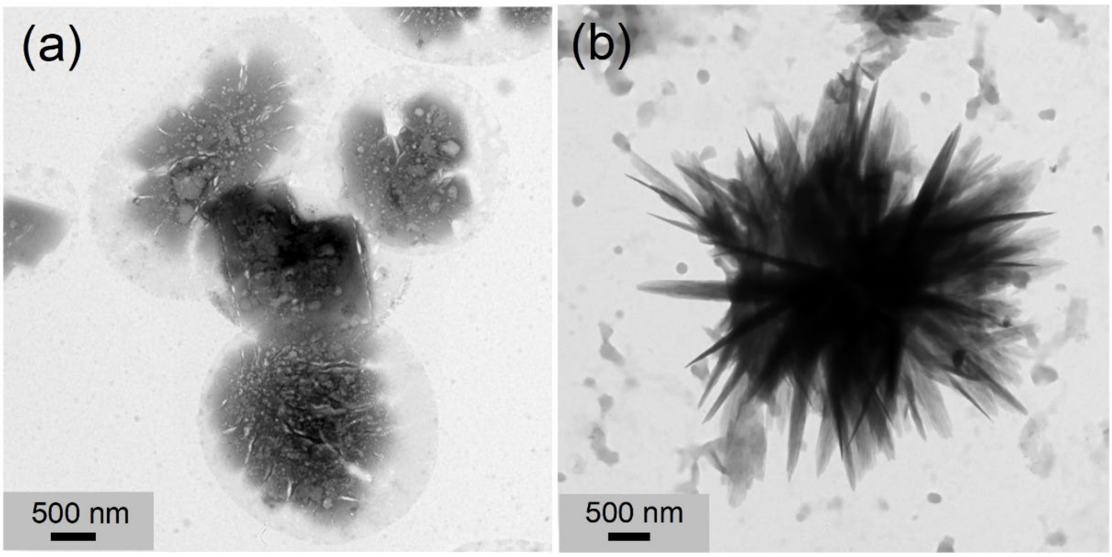

Figure S8. Ni-OAm-$NaBH_4$ at (a) RT after 18 h and (b) 80 °C after 1 h.

9. XRD pattern of pristine $NaBH_4$

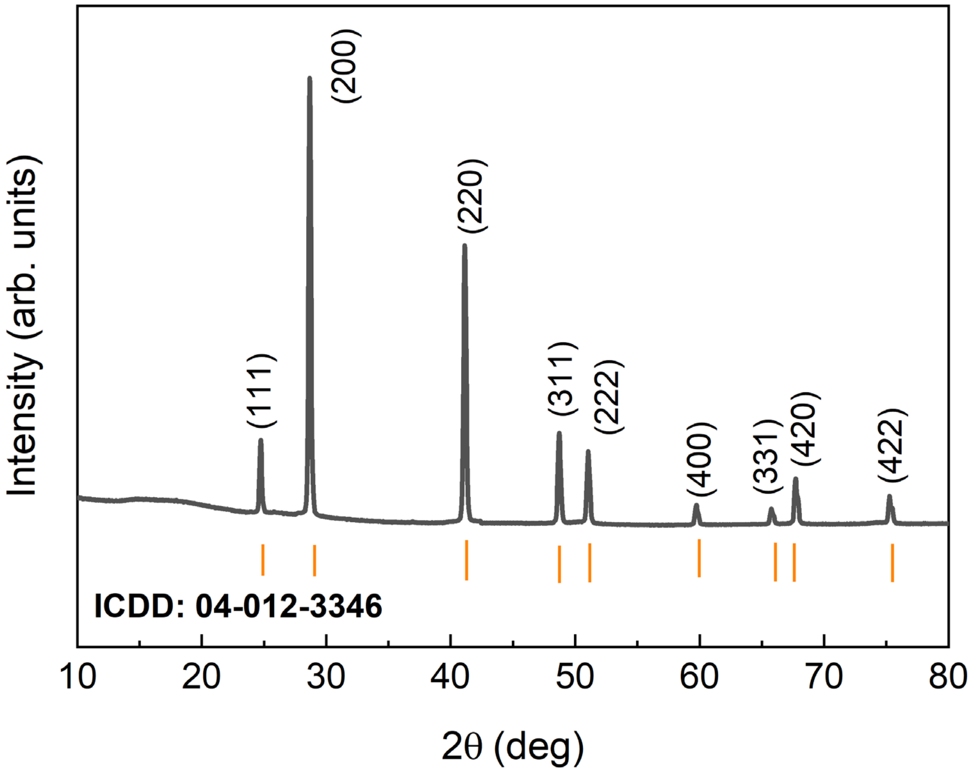

Figure S9. XRD pattern of bulk $NaBH_4$. The XRD pattern was retrieved from International Centre for Diffraction Data (ICDD).

10. FTIR spectra of pristine $NaBH_4$

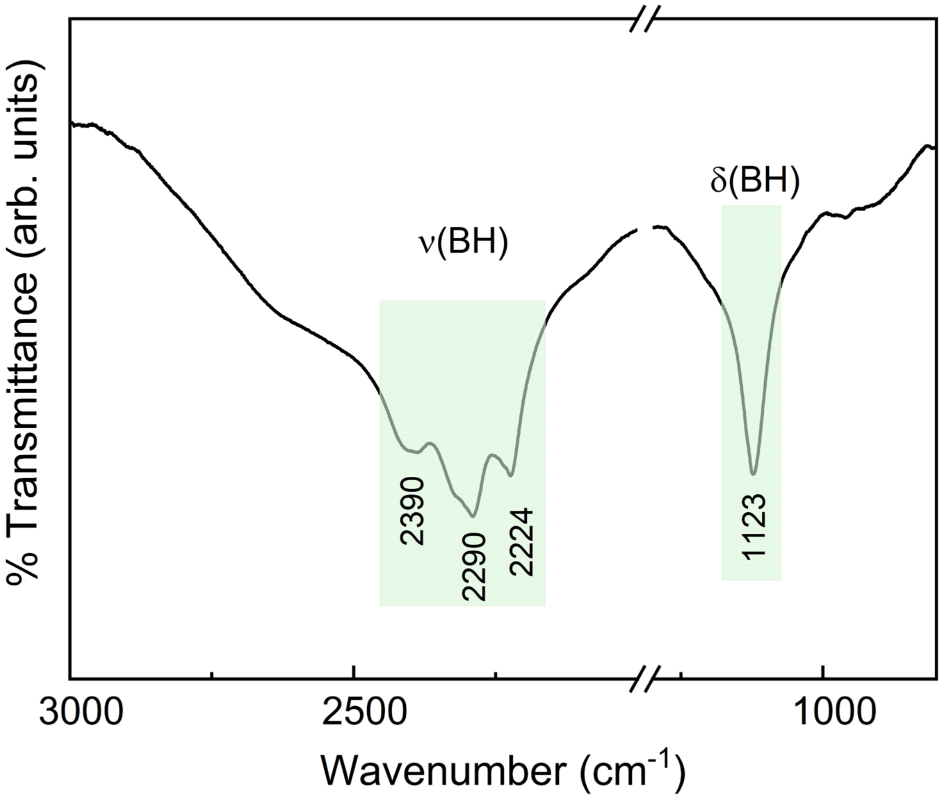

Figure S10. FTIR spectrum of pristine $NaBH_4$.

11. XRD intensity comparison of $NaBH_4$-TBAB and $NaBH_4$-TBAB@Ni

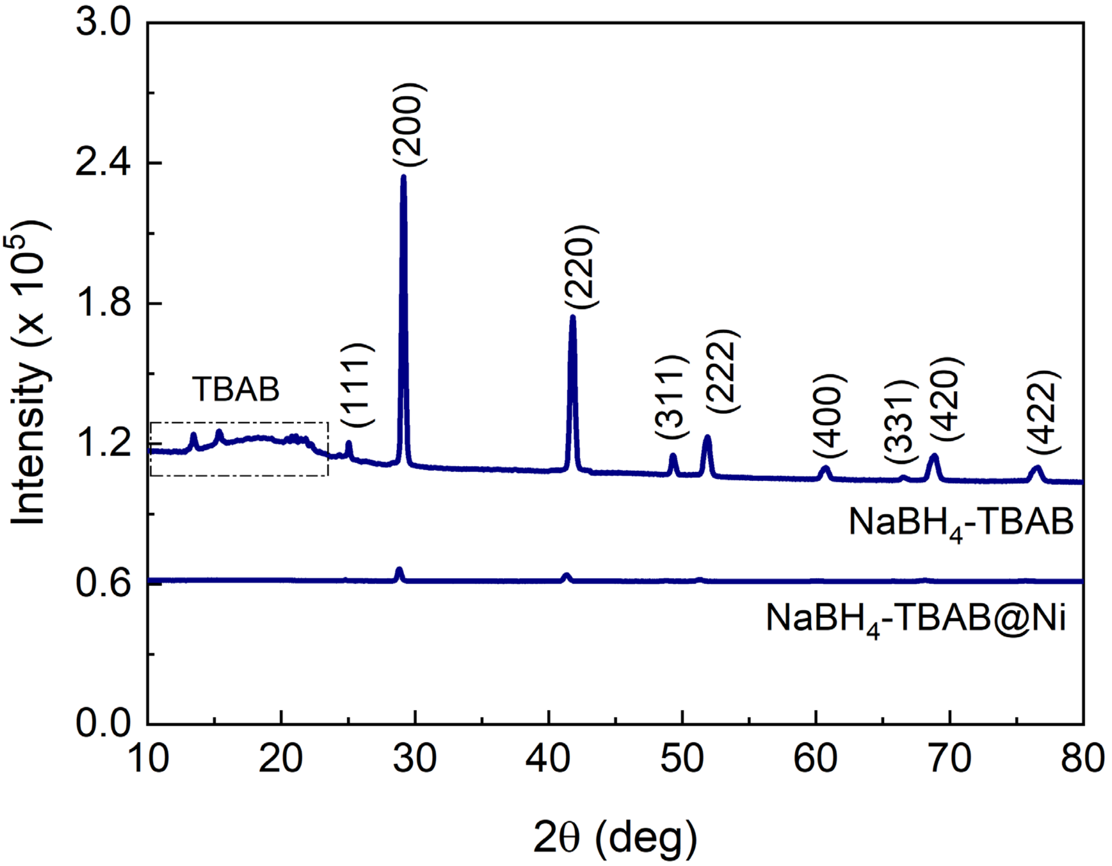

Figure S11. Comparison of XRD intensity of $NaBH_4$-TBAB (before Ni coating) and $NaBH_4$-TBAB@Ni (after Ni coating).

12. XRD intensity comparison of $NaBH_4$-ODA and $NaBH_4$-ODA@Ni

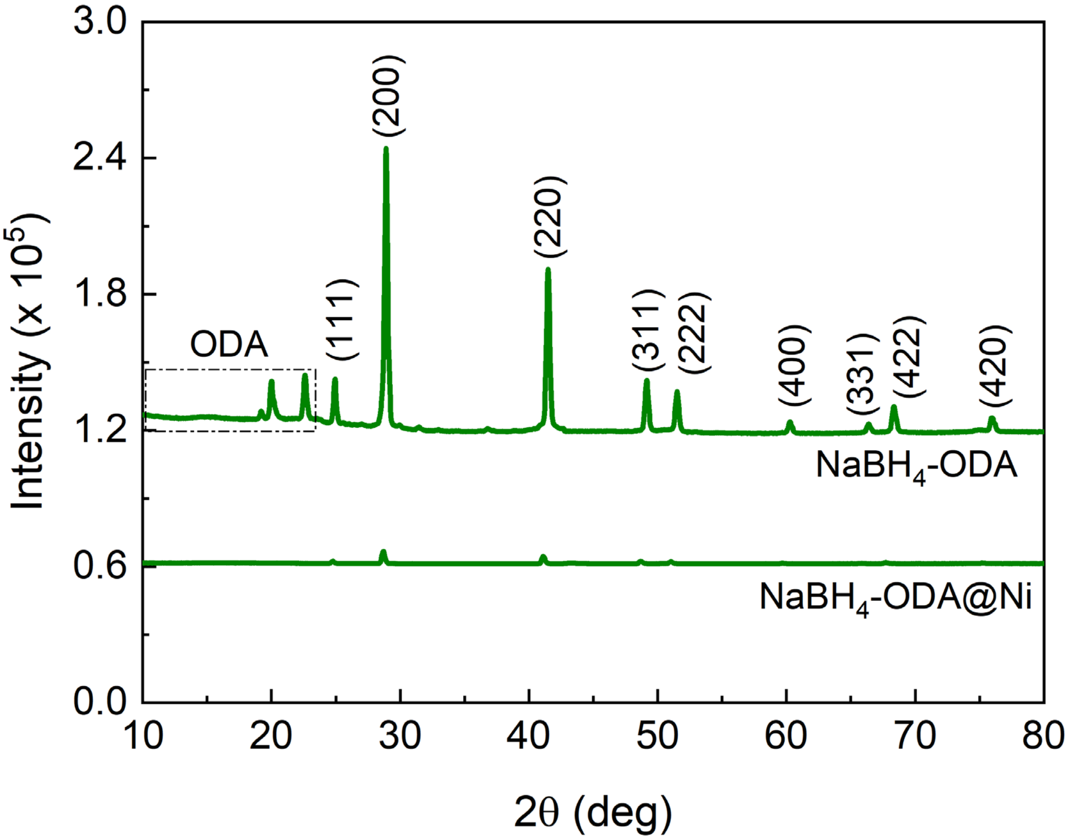

Figure S12. Comparison of XRD intensity of $NaBH_4$-ODA (before Ni coating) and $NaBH_4$-ODA@Ni (after Ni coating).

13. XRD intensity comparison of $NaBH_4$-TDA and $NaBH_4$-TDA@Ni

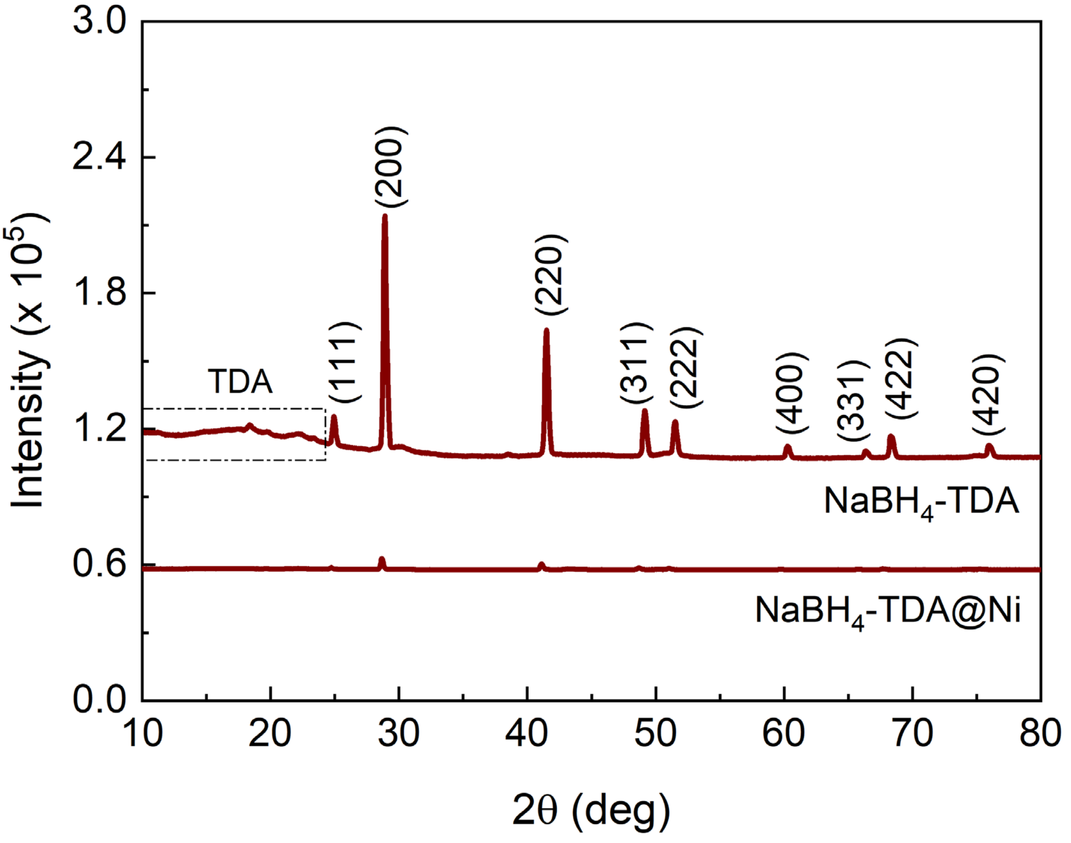

Figure S13. Comparison of XRD intensity of $NaBH_4$-TDA (before Ni coating) and $NaBH_4$-TDA@Ni (after Ni coating).

14. XPS spectra of NaBH4-TBAB@Ni

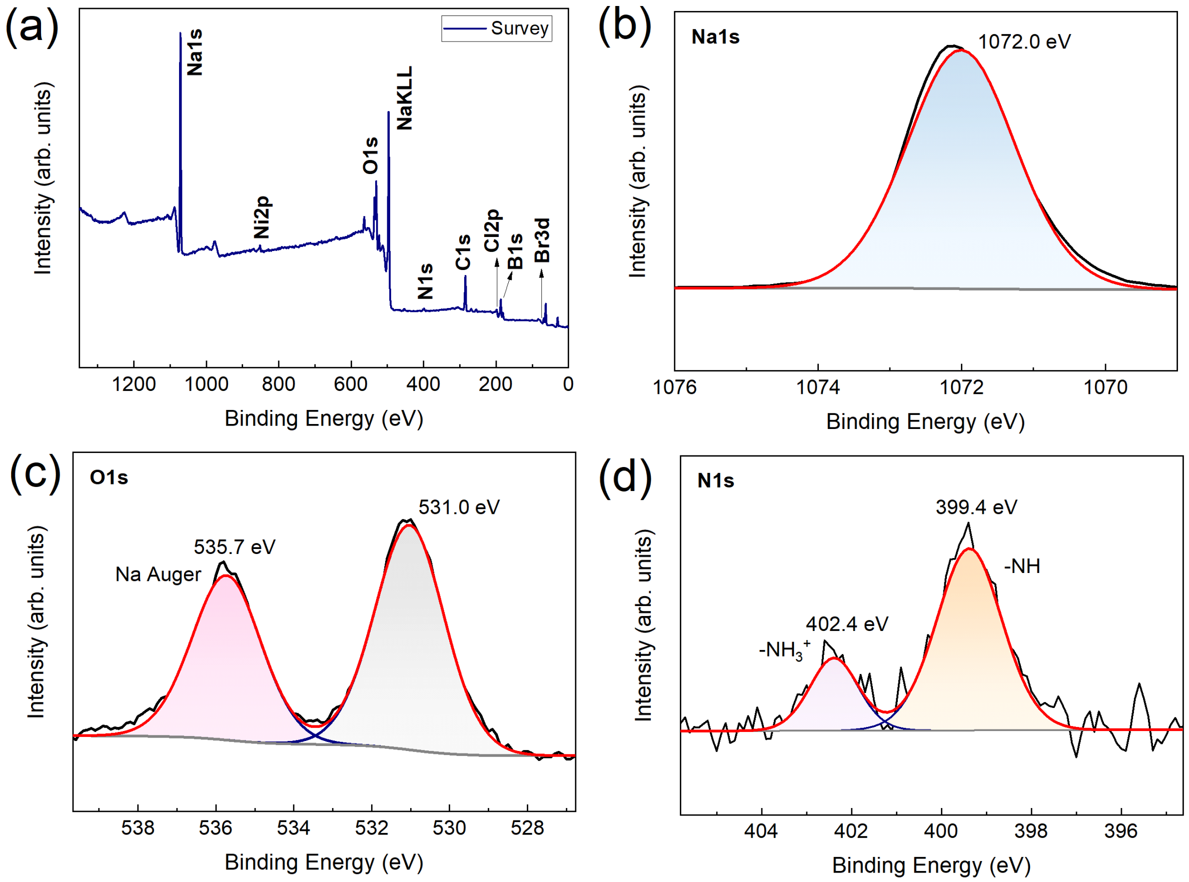

Figure S14. (a) XPS survey scan and (b–d) high-resolution spectra (Na1s, O1s, N1s) of NaBH4-TBAB@Ni. The Cl2p at 198 eV in (a) could be due to chloride ions from the Ni-OAm complex.

15. XPS spectra of $NaBH_4$-ODA@Ni

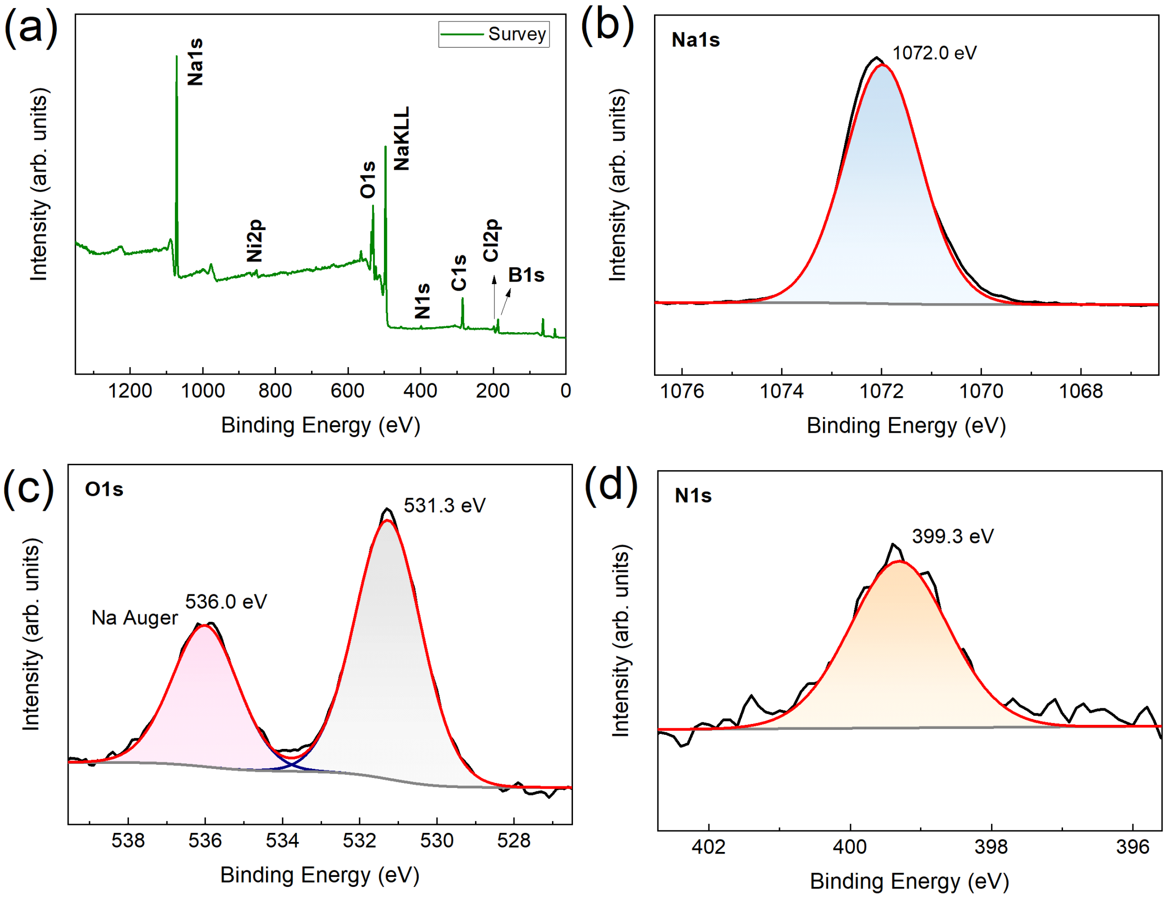

Figure S15. (a) XPS survey scan and (b–d) high-resolution spectra (Na1s, O1s, N1s) of $NaBH_4$-ODA@Ni. The Cl2p at 198 eV in (a) could be due to chloride ions from the Ni-OAm complex.

16. XPS spectra of $NaBH_4$-TDA@Ni

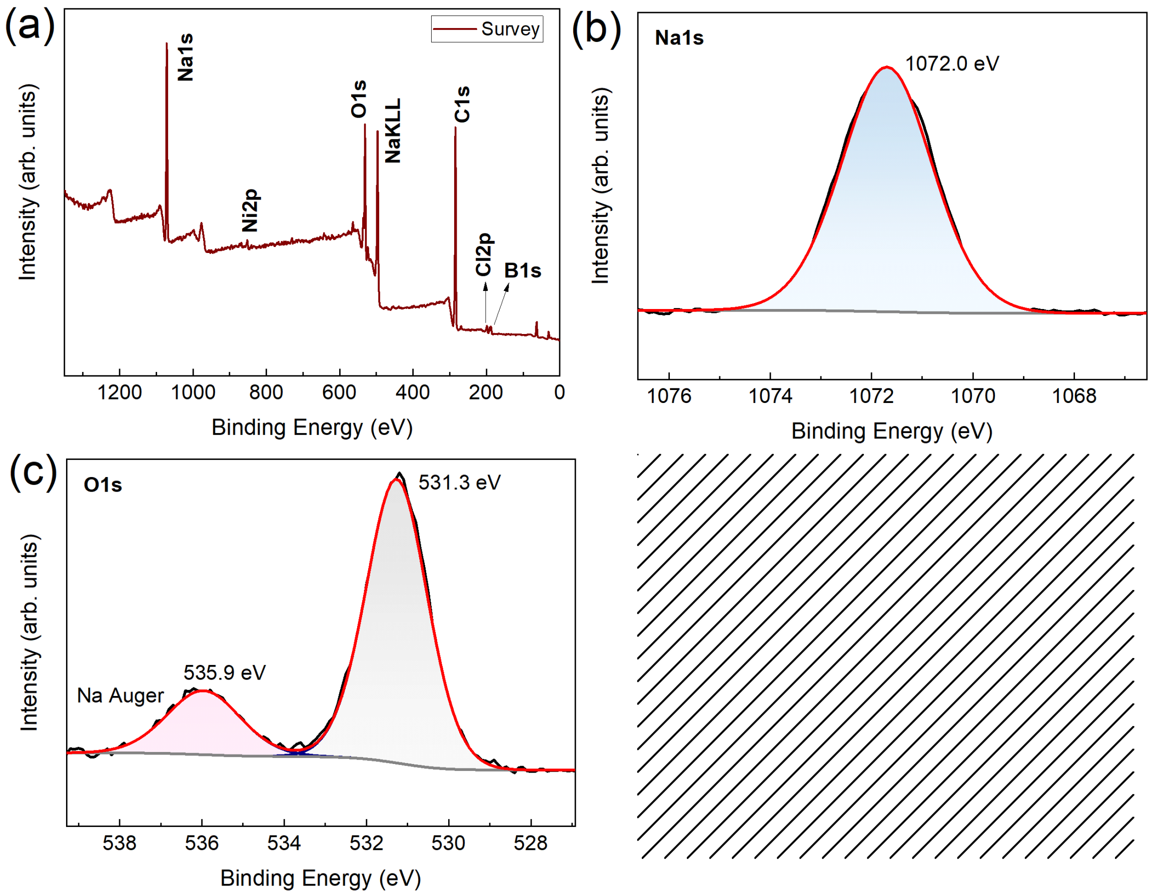

Figure S16. (a) XPS survey scan and (b, c) high-resolution spectra (Na1s, O1s)) of $NaBH_4$-TDA@Ni. The Cl2p at 198 eV in (a) could be due to chloride ions from the Ni-OAm complex.

17. XPS spectra of pristine $NaBH_4$

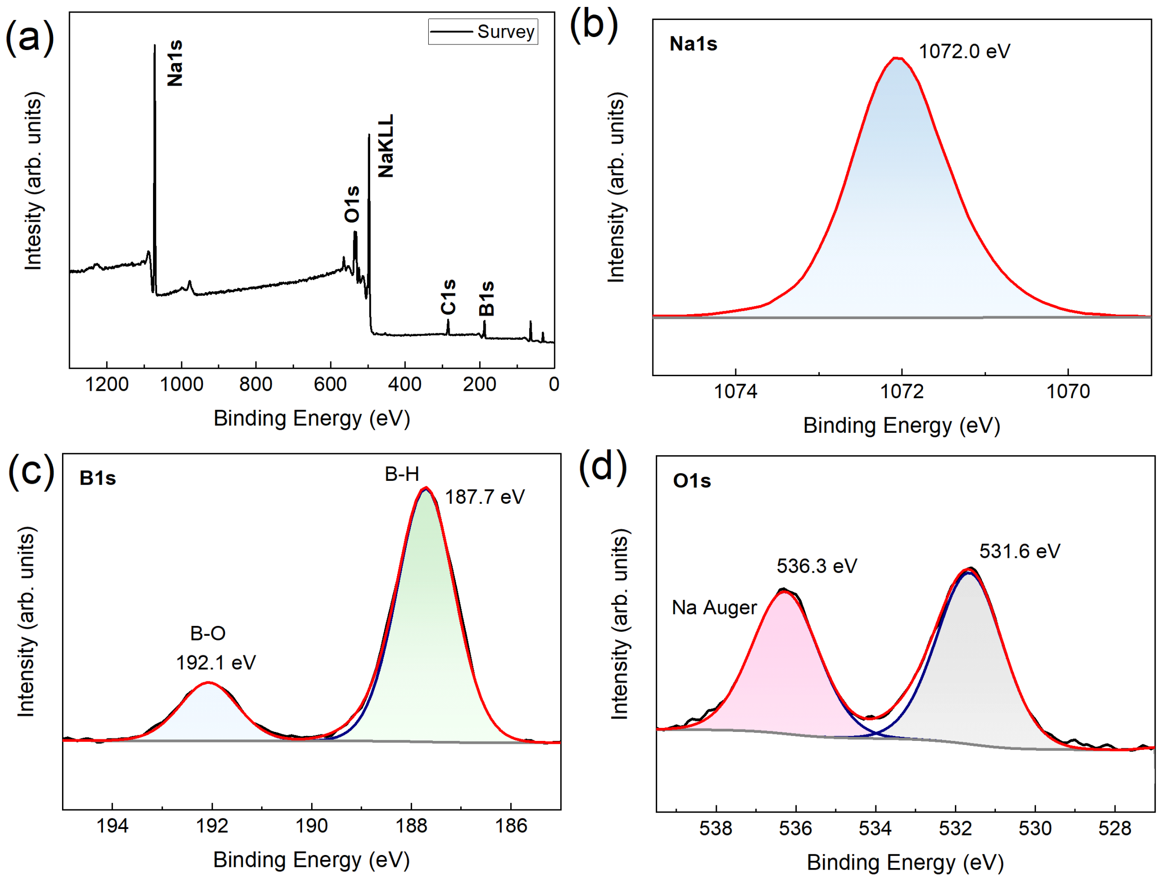

Figure S17. (a) XPS survey scan (b–d) high-resolution spectra (Na1s, B1s, O1s) of pristine $NaBH_4$.

Explanation of Figures S14-S17

The XPS survey scan revealed the presence of Na, B, Ni, N, Br, O and C elements in $NaBH_4$@Ni (Figures S14–S16). For $NaBH_4$-TBAB@Ni, the high-resolution XPS spectra of the selected elements are given in Figure S14b-e. The Na1s peak at 1072.0 eV is attributed to $NaBH_4$ in $NaBH_4$-TBAB@Ni.[2] The O1s peak located at 531.0 eV indicates the presence of the B-O-Ni bonds[3] and/or defect sites with low oxygen coordination.[4] We also noted that even after multiple washings of $NaBH_4$-TBAB@Ni there were traces of TBAB surfactant left

(e.g. N and Br elements), which could be due to the interaction between the TBAB and $NaBH_4$. Figure S14d shows the presence of -NH from butylammonium group of TBAB. The presence of Br (located at 182–189 eV) is evidenced by the XPS survey scan for Br3d (Figure S14a, high-resolution XPS spectra are not shown).

For $NaBH_4$-ODA@Ni, the XPS survey scan revealed the presence of Na, B, Ni, N, O and C elements (Figure S15a). Whereas for $NaBH_4$-TDA@Ni, the survey scan shows the presence of Na, B, Ni, O and C (Figure S16a). The high-resolution XPS spectra of Na, O, N, and C and Na, O, C for $NaBH_4$-ODA@Ni and $NaBH_4$-TDA@Ni (Figures S15b-e and S16b-c), respectively, show peak positions similar to $NaBH_4$-TBAB@Ni (Figure S14b). Once again, the presence of N indicates the interaction of $NaBH_4$ and ODA, and thus the presence of -NH_2 groups on the surface of $NaBH_4$. Moreover, the XPS spectra of Na, O, and C of the core materials (Figures S14–S16) are comparable to the pristine $NaBH_4$ (Figure S17). For all the materials, the C1s could be ascribed to the adventitious carbon.[5]

18. Simulated NEXAFS spectra

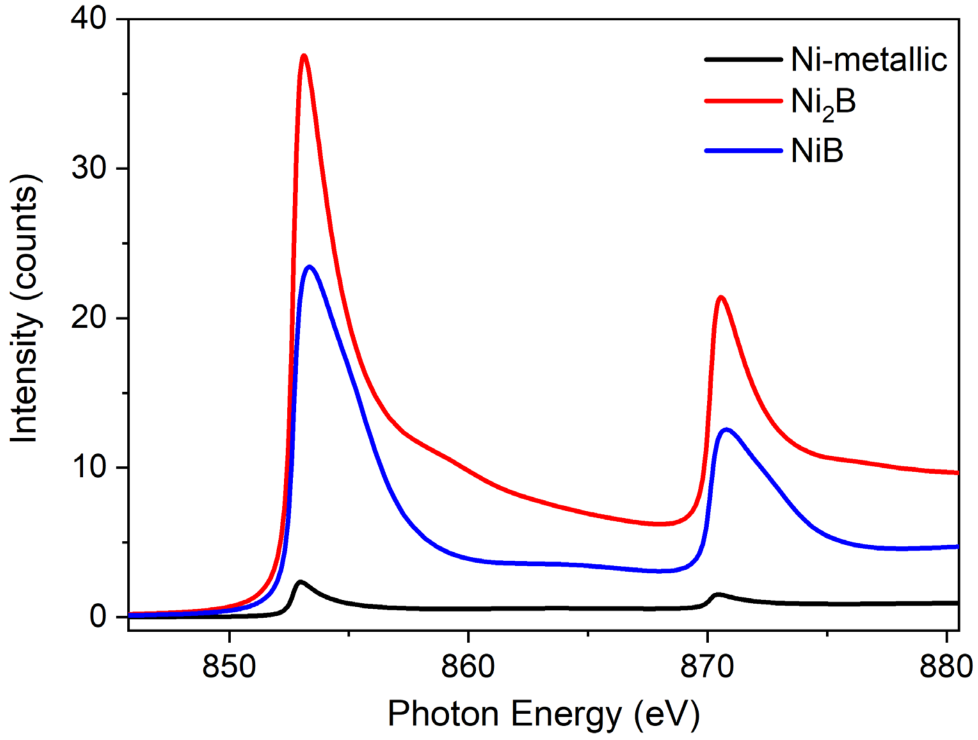

Figure S18. Intensity comparison of the simulated NEXAFS spectra of NiB and Ni_2B with Ni metallic.

19. Island growth of Ni shell at RT

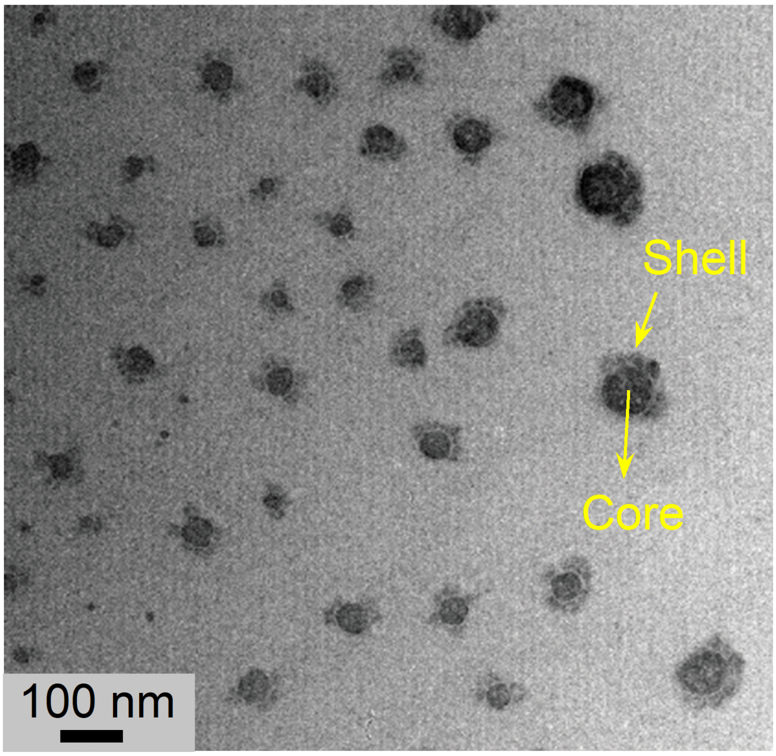

Figure S19. Nonuniform Ni shell in core-shell $NaBH_4$-TBAB@Ni obtained at RT after 18 h in toluene.

20. Core-shell $NaBH_4$@Ni in cyclohexane with discrete Ni particles

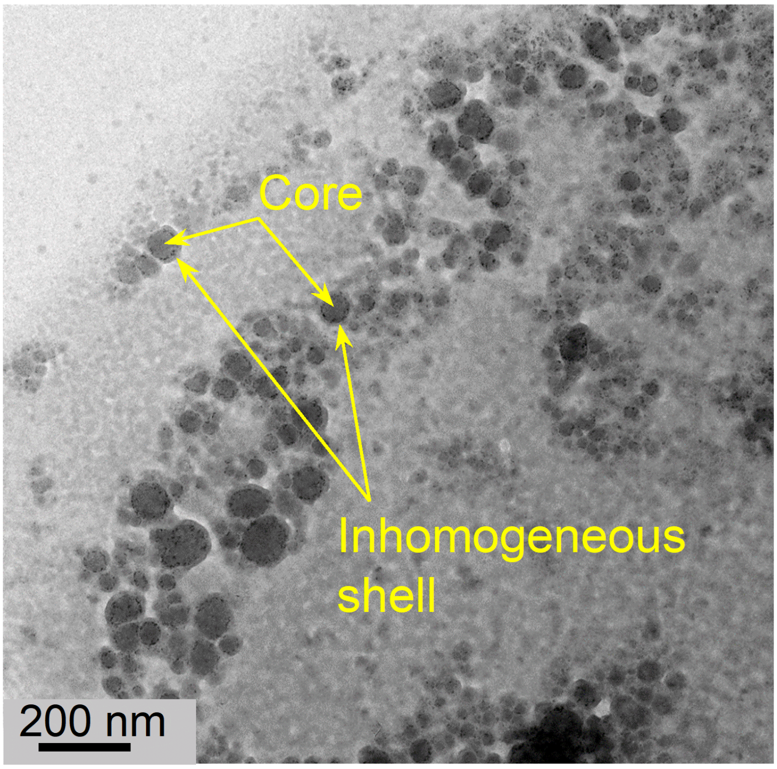

Figure S20. Core-shell $NaBH_4$-TBAB@Ni particles obtained at RT after 18 h in cyclohexane.

21. Primary core-shell particles

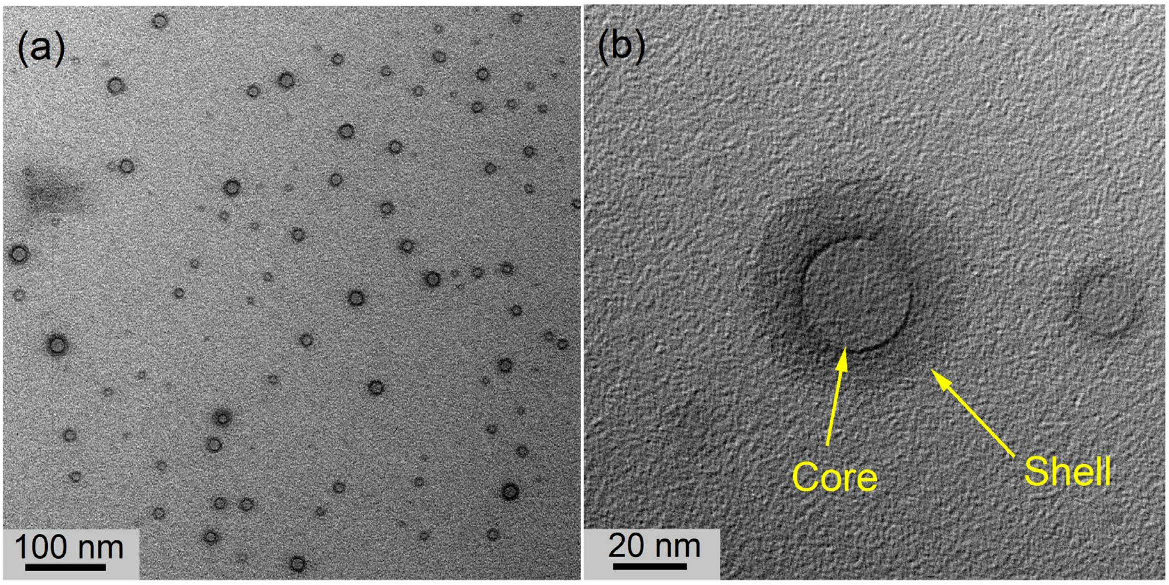

Figure S21. TEM images (a and b) of primary $NaBH_4$-TBAB@Ni core-shell particles at 40 °C. The core of the core-shell particle in (b) behaves like a trapped bubble inside a shell, which can be seen in a movie clip (Supporting Video S1).

22. Isolated Ni particles with core-shell $NaBH_4$-TBAB@Ni at 80 °C

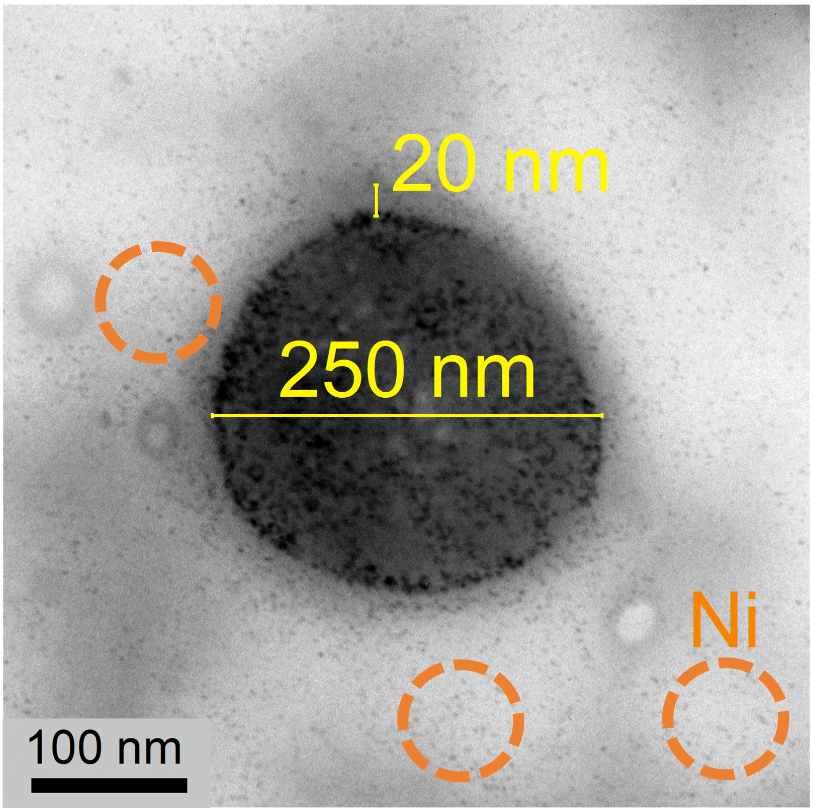

Figure S22. TEM image of core-shell $NaBH_4$-TBAB@Ni particle surrounded by the isolated Ni particles obtained at 80 °C, 3 h.

23. Core-shell $NaBH_4$-ODA@Ni at different temperatures and times

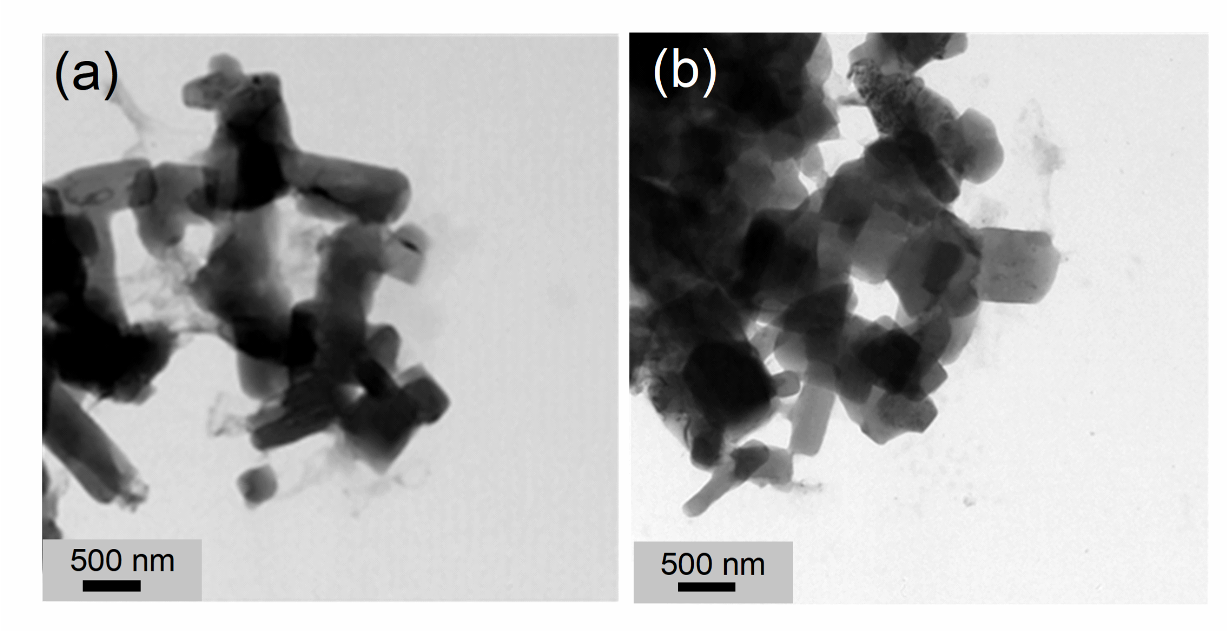

Figure S23. TEM of core-shell $NaBH_4$-ODA@Ni particles obtained after (a) 3 h and (b) 6 h in toluene at 40 °C.

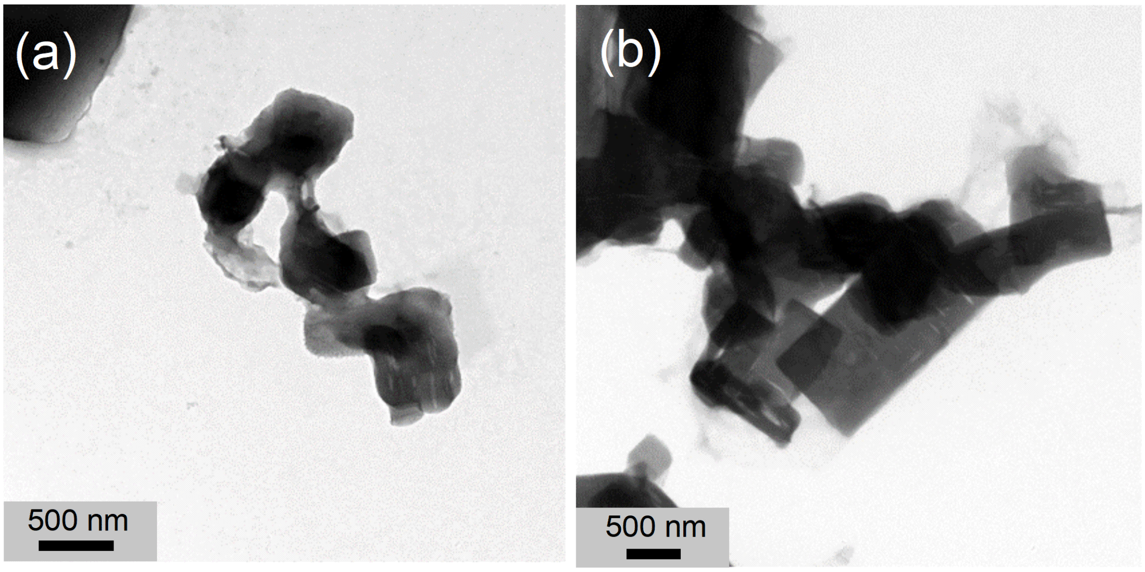

Figure S24. TEM of core-shell $NaBH_4$-ODA@Ni particles obtained after (a) 3 h and (b) 6 h in toluene at 60 °C.

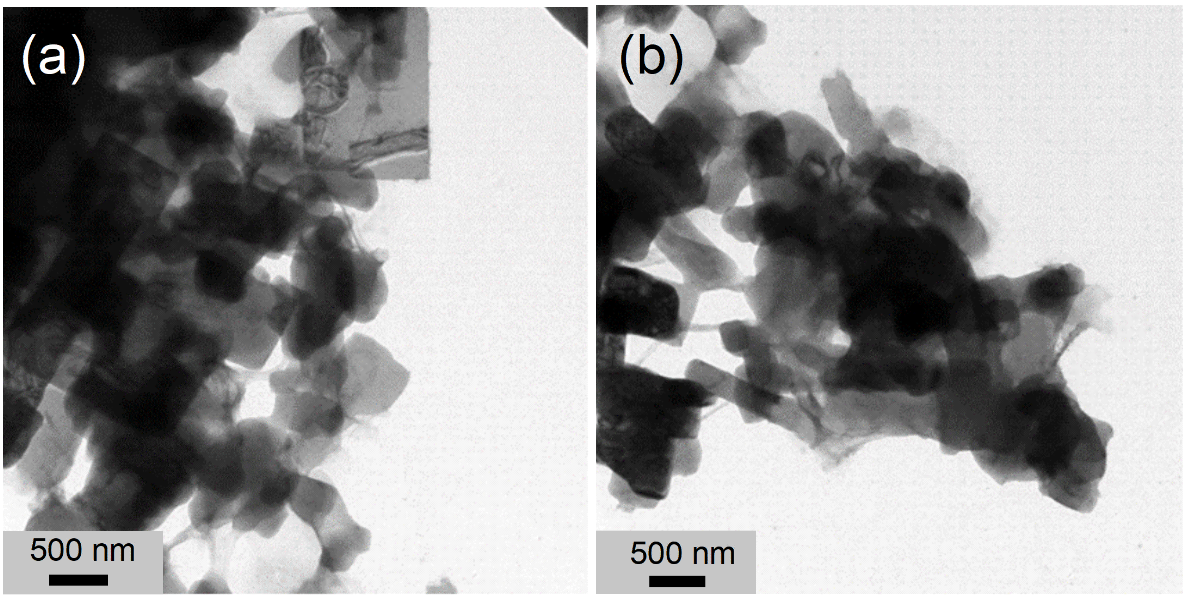

Figure S25. TEM of core-shell $NaBH_4$-ODA@Ni particles obtained after (a) 3 h and (b) 6 h in toluene at 80 °C.

24. Core-shell $NaBH_4$-TDA@Ni at different temperatures and times

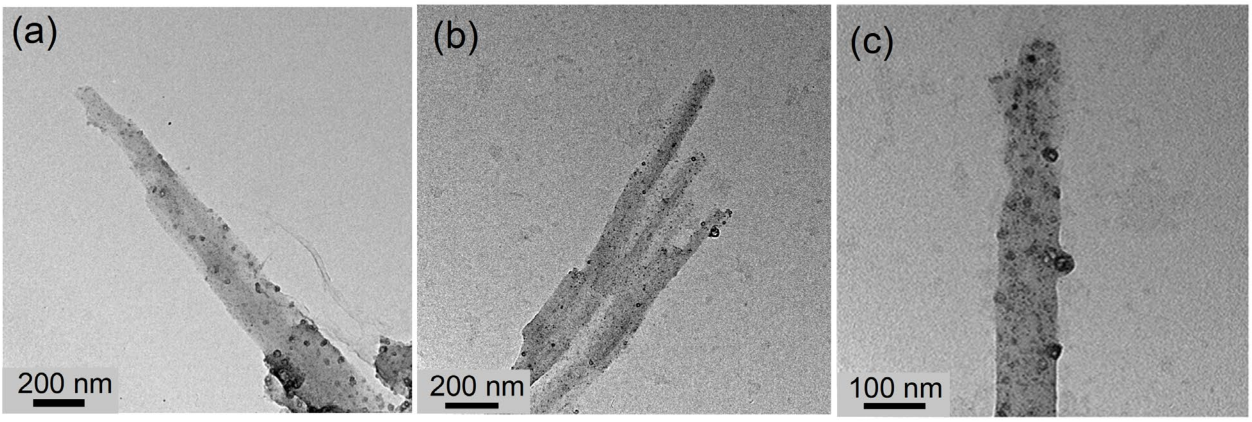

Figure S26. TEM of core-shell $NaBH_4$-TDA@Ni obtained after (a) 3 h, (b) 6 h and (c) 18 h at 40 °C.

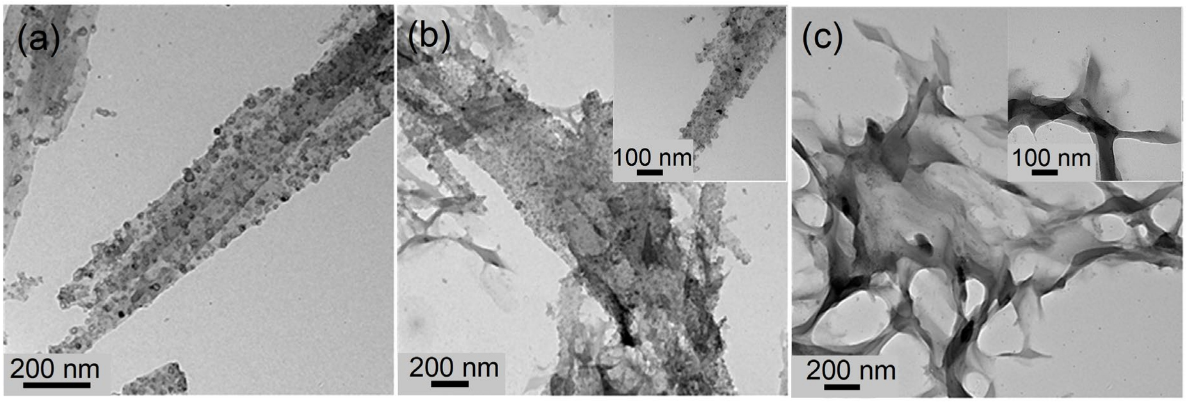

Figure S27. Core-shell $NaBH_4$-TDA@Ni obtained after (a) 3 h, (b) 6 h and (c) 18 h at 60 °C.

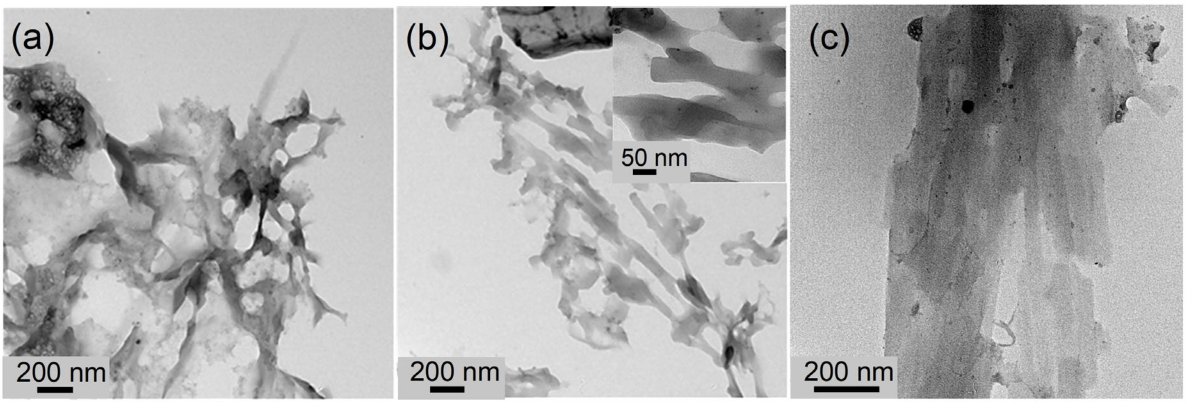

Figure S28. Core-shell $NaBH_4$-TDA@Ni obtained after (a) 3 h, (b) 6 h, and (c) 18 h at 80 °C.

Explanation of Figures S23–S28

Figures S23–S25 show the TEM images of the core-shell $NaBH_4$-ODA@Ni structures obtained at 40, 60 and 80 °C after 3–6 h. After 3–6 h (Figures S23–S25), the morphology of the core-shell structure remained stable signifying good structural integrity during the synthesis. Figure S26 shows the TEM images of the core-shell structures obtained between 3–18 h at 40 °C using $NaBH_4$-TDA. The island growth of Ni particles on the bars was observed even after extending the aging time i.e. after 6–18 h (Figure S26b, c). At 60 °C, the density of the Ni particles increased on the $NaBH_4$ bars during 3–6 h (Figure S27). This indicates that the Ni-OAm was deposited and reduced to the Ni species and the resultant

core-shell structures exhibited good stability at 60 °C (up to 6 h). However, after 18 h at 60 °C, the core-shell showed severe agglomeration and isolated Ni particles can be seen around the $NaBH_4$ bars indicating that the structure collapsed after prolonged stirring (Figure S27c). Similar results were obtained at 80 °C, where the bars were severely agglomerated and the density of the Ni particles on the bars decreased during aging (Figure S28).

25. TGA/DSC and hydrogen release profiles of core-shell nanoarchitectures

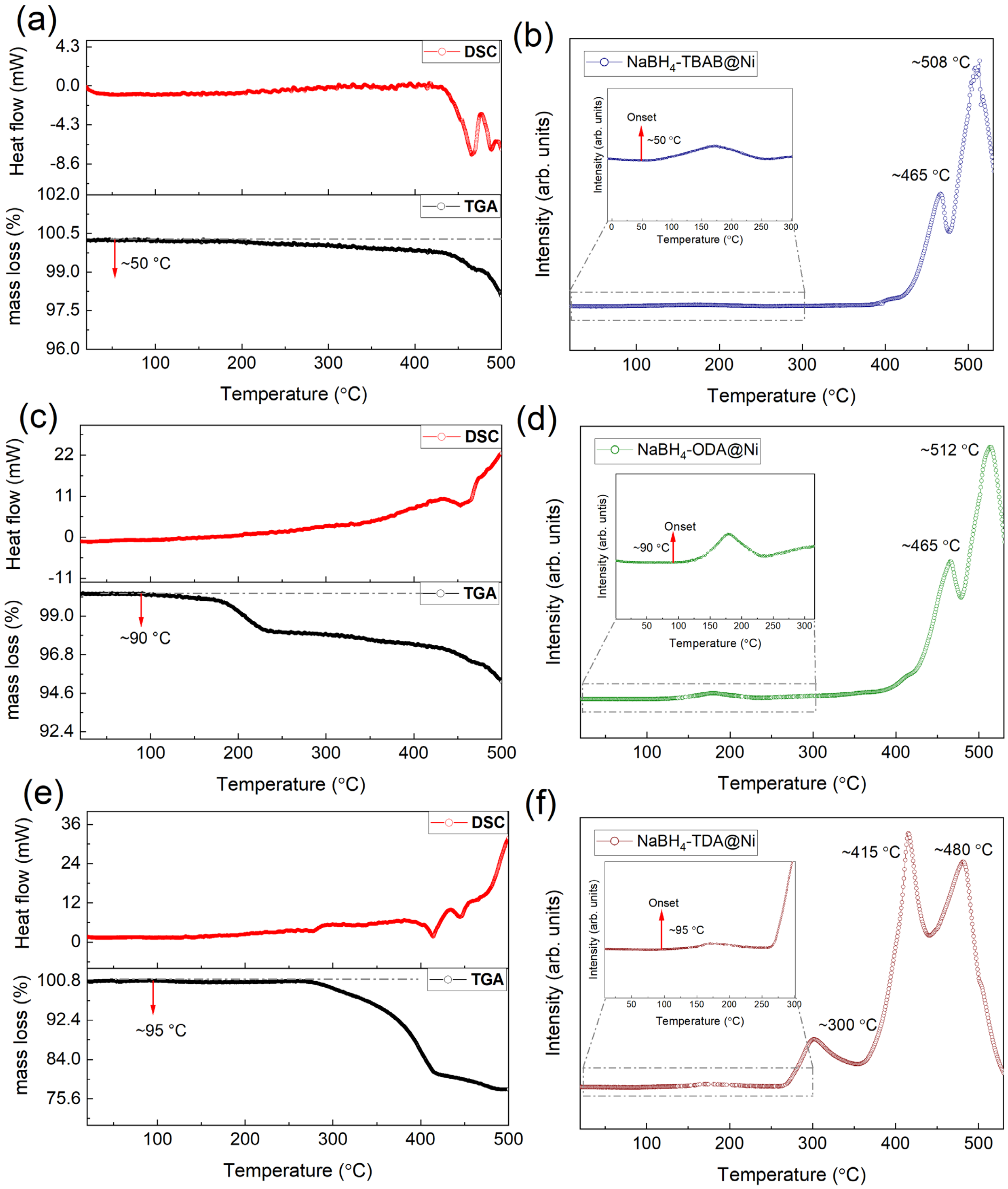

Figure S29. TGA/DSC (a, c, e) and MS (b, d, f) for $NaBH_4$-TBAB@Ni, $NaBH_4$-ODA@Ni and $NaBH_4$-TDA@Ni. The initial (onset) temperature for mass loss and hydrogen release has been indicated (red arrow). Only hydrogen was detected in MS (b, d, f).

Supporting Videos

1. Video S1

Primary core-shell $NaBH_4$@Ni particle under TEM.

2. Video S2

The video S2 shows the decomposition of $NaBH_4$ from a representative core-shell $NaBH_4$-TBAB@Ni particle after prolonged exposure under the electron beam in TEM. The Ni shell (dark) is visible after the decomposition of the $NaBH_4$ core.

References

[1] S. Carenco, C. Boissière, L. Nicole, C. Sanchez, P. Le Floch, N. Mézailles, *Chem. Mater.* **2010**, *22*, 1340-1349.

[2] M. L. Christian, K.-F. Aguey-Zinsou, *ACS Nano* **2012**, *6*, 7739-7751.

[3] a) J. Legrand, S. Gota, M. J. Guittet, C. Petit, *Langmuir* **2002**, *18*, 4131-4137; b) W.-J. Jiang, S. Niu, T. Tang, Q.-H. Zhang, X.-Z. Liu, Y. Zhang, Y.-Y. Chen, J.-H. Li, L. Gu, L.-J. Wan, J.-S. Hu, *Angew. Chem. Int. Ed.* **2017**, *56*, 6572-6577.

[4] a) R. Chen, H.-Y. Wang, J. Miao, H. Yang, B. Liu, *Nano Energy* **2015**, *11*, 333-340; b) V. M. Jiménez, A. Fernández, J. P. Espinós, A. R. González-Elipe, *J. Electron. Spectrosc. Relat. Phenom.* **1995**, *71*, 61-71; c) C. An, Y. Wang, Y. Huang, Y. Xu, C. Xu, L. Jiao, H. Yuan, *CrystEngComm* **2014**, *16*, 385-392.

[5] H.-K. Jeong, Y. P. Lee, R. J. W. E. Lahaye, M.-H. Park, K. H. An, I. J. Kim, C.-W. Yang, C. Y. Park, R. S. Ruoff, Y. H. Lee, *J. Am. Chem. Soc.* **2008**, *130*, 1362-1366.
